# Comparative Genomics of Typical and Atypical *Aeromonas salmonicida* Complete Genomes Revealed New Insights into Pathogenesis Evolution

**DOI:** 10.3390/microorganisms10010189

**Published:** 2022-01-15

**Authors:** Ignacio Vasquez, Ahmed Hossain, Hajarooba Gnanagobal, Katherinne Valderrama, Briony Campbell, Michael Ness, Steve J. Charette, Anthony K. Gamperl, Rocco Cipriano, Cristopher Segovia, Javier Santander

**Affiliations:** 1 Marine Microbial Pathogenesis and Vaccinology Laboratory, Department of Ocean Sciences, Memorial University of Newfoundland, 0 Marine Lab Rd., St. John’s, NL A1C 5S7, Canada; ivasquezsoli@mun.ca (I.V.); ahossain@mun.ca (A.H.); hgnanagobal@mun.ca (H.G.); kvalderrama@mun.ca (K.V.); cwsegovia@mun.ca (C.S.); 2 Golden Eagle Sable Fish 4.7. 335 Walkers Hook Rd., Salt Spring Island, BC V8K 1P5, Canada; briony.campbell@goldeneaglesablefish.com; 3 PharmaQ, 0275 Oslo, Norway; michael.ness@zoetis.com; 4 Department of Biochemistry, Microbiology and Bioinformatics, Faculty of Science and Engineering, Laval University, Quebec, QC G1V 0A6, Canada; steve.charette@bcm.ulaval.ca; 5 Fish Physiology Laboratory, Department of Ocean Science, Memorial University of Newfoundland, St John´s, NL A1C 5S7, Canada; kgamperl@mun.ca; 6 U.S. Geological Survey (USGS), National Fish Health Research Laboratory, Kearneysville, WV 25430, USA; rocco.cipriano@yahoo.com

**Keywords:** *Aeromonas salmonicida*, genomics, taxonomy, virulence, plasmidome, insertion sequences

## Abstract

*Aeromonas salmonicida* is a global distributed Gram-negative teleost pathogen, affecting mainly salmonids in fresh and marine environments. *A. salmonicida* strains are classified as typical or atypical depending on their origin of isolation and phenotype. Five subspecies have been described, where *A.* *salmonicida* subsp. *salmonicida* is the only typical subspecies, and the subsp. *achromogenes*, *masoucida*, *smithia*, and *pectinolytica* are considered atypical. Genomic differences between *A. salmonicida* subsp. *salmonicida* isolates and their relationship with the current classification have not been explored. Here, we sequenced and compared the complete closed genomes of four virulent strains to elucidate their molecular diversity and pathogenic evolution using the more accurate genomic information so far. Phenotypes, biochemical, and enzymatic profiles were determined. PacBio and MiSeq sequencing platforms were utilized for genome sequencing. Comparative genomics showed that atypical strains belong to the subsp. *salmonicida,* with 99.55% ± 0.25% identity with each other, and are closely related to typical strains. The typical strain *A. salmonicida* J223 is closely related to typical strains, with 99.17% identity with the *A.* *salmonicida* A449. Genomic differences between atypical and typical strains are strictly related to insertion sequences (ISs) activity. The absence and presence of genes encoding for virulence factors, transcriptional regulators, and non-coding RNAs are the most significant differences between typical and atypical strains that affect their phenotypes. Plasmidome plays an important role in *A. salmonicida* virulence and genome plasticity. Here, we determined that typical strains harbor a larger number of plasmids and virulence-related genes that contribute to its acute virulence. In contrast, atypical strains harbor a single, large plasmid and a smaller number of virulence genes, reflected by their less acute virulence and chronic infection. The relationship between phenotype and *A. salmonicida* subspecies’ taxonomy is not evident. Comparative genomic analysis based on completed genomes revealed that the subspecies classification is more of a reflection of the ecological niche occupied by bacteria than their divergences at the genomic level except for their accessory genome.

## 1. Introduction

*Aeromonas salmonicida* is one of the oldest fish pathogens known and the causative agent of furunculosis in a wide range of hosts that live in freshwater and marine systems [1,2]. *A. salmonicida* classification is based on the origin of isolation. *A. salmonicida* strains isolated from salmonids are classified as “typical” and strains isolated from non-salmonid hosts or environmental samples are classified as “atypical” strains [1,2,3].

*A. salmonicida* includes five subspecies; *A. salmonicida* subsp. *salmonicida* is the only typical subspecies, whereas the subsp. *masoucida*, *smithia*, *achromogenes*, and *pectinolytica* are considered atypical [1,4]. Previous classification and phylogenetic studies of *A. salmonicida* isolates have demonstrated that there is high genomic divergency and plasticity among subspecies [5]. This diversity is also reflected in the different biochemical features, colony size, growth rate, brown pigment synthesis, hemolytic activity, and origin of host isolation [6,7,8]. However, these characteristics are often inconsistent with the current classification. For instance, the optimal growth temperature is used to distinguish between typical and atypical *A. salmonicida* strains. *A. salmonicida* subsp. *salmonicida*, *achromogenes*, *masoucida*, and *smithia* strains are considered psychrotrophs, in contrast to *pectinolitytica* subspecies, which are considered mesophilic [5]. However, endogenous transposition mutagenesis can generate mesophilic strains from psychrotroph strains or vice versa [9]. The *vapA* gene, which encodes the protein found in the A-layer, is highly unstable in some strains of *A. salmonicida* and susceptible to endogenous transposition mutagenesis [10], but it is also contradictorily utilized for *A. salmonicida* subsp. classification [11].

Previous genomic studies have described that *A. salmonicida* genome plasticity and divergency is strictly related to mobile genetic element activity, driving the chromosomal re-arrangements involved in environmental adaptation [8], pathogenesis, and host range [12]. However, most of these studies were performed using short-reads assembled genome sequences, displaying limitations associated with the high number of contigs generated [13,14]. For instance, it was documented in *A. salmonicida* that the activity of ISs causes a loss of virulence [15] and mutations in genes involved in lipopolysaccharide biogenesis and in the A-layer-encoding genes [9,16]. Recently, it has been described that the plasmidome of *A. salmonicida* highly relates with the typical and atypical phenotype and their taxonomy, while the chromosome sequence remains highly similar between subspecies [14,17]. The classification of *A. salmonicida* subspecies based on the previously mentioned genetic analysis and their phenotypic relationship is not optimal, which suggests that having multiple closed complete *A. salmonicida* genomes and comparing them in a whole genome base could provide access to more accurate information about mobile genetic elements and chromosomal re-arrangements.

Non-coding RNAs (ncRNAs) play essential roles in many biological processes, such as signaling pathways, gene regulation, RNA processing, and protein synthesis, including virulence [18,19]. Initially, ncRNAs were identified in *Escherichia coli* [18,19,20]. Nowadays, ncRNAs have been identified in several bacterial and archaeal species [21,22], including marine pathogens, such as *Piscirickettsia salmonis* [23], *Vibrio salmonicida* [24], and *Aliivibrio salmonicda* [25], but annotation of ncRNAs in *A. salmonicida* has not been documented.

In this study, we conducted comparative phenotypic and genomic analyses between *A. salmonicida* typical and atypical strains isolated from Atlantic salmon (*Salmo salar*) and sablefish (*Anoplopoma fimbria*), respectively, taking advantage of long-read and short-read sequencing methods to perform the first side-by-side genomic analysis of multiple close genomes of *A. salmonicida*. This analysis indicated that differences within typical and atypical strains could be mainly attributed to the number of plasmids, chromosomal re-arrangements, the absence of the type 3 secretion system (T3SS) injectosome and effectors, insertion sequences (ISs) copy number, and non-coding RNAs.

## 2. Material and Methods

### 2.1. Bacterial Characterization

#### 2.1.1. *Aeromonas salmonicida* Strains 

The pathogenic atypical *A. salmonicida* strains J409, J410, and J411 were isolated from the head kidney of three infected adult sablefish from net pens in the Kyoquot Sound, British Columbia, Canada. Typical *A. salmonicida* J223 was isolated from Atlantic salmon in 1999 [26]. Bacterial strains were grown in Trypticase Soy Agar (TSA) supplemented with Congo red (50 μg/mL; Sigma) plates to verify the presence of the A-layer [27]. *A. salmonicida* strains were cryopreserved at −80 °C in glycerol 10% and peptone 1% solution.

#### 2.1.2. Bacterial Culture Conditions

*A. salmonicida* strains were grown routinely from a single colony in 3 mL of Trypticase Soy Broth (TSB, Difco, Franklin Lakes, NJ) at 15 °C in a 16 mm diameter glass tube and placed in a roller drum (TC7, New Brunswick, MA, USA) for 24 h. When required, the culture media were supplemented with 100 µM of FeCl_3_ (100 mM), 2,2-dipyridyl (100 mM), or 1.5% bacto-agar (Difco). Chrome azurol-S (CAS) plates were used to evaluate siderophores secretion [28]. Blood agar plates (5% salmon blood and 5% sheep blood) were used to evaluate hemolysis activity. Bacterial cells were harvested at the mid-log phase and an optical density (OD_600 nm_) of ~0.7 (~6.3 × 10^8^ CFU/mL), and they were washed three times with phosphate-buffered saline (PBS; 136 mM NaCl, 2.7 mM KCl, 10.1 mM Na_2_HPO_4_, and 1.5 mM KH_2_PO_4_ (pH 7.2)) [29] at 4200× *g* for 10 min at room temperature before analysis.

#### 2.1.3. Phenotypic, Biochemical, and Enzymatic Testing

To determine the physiological differences between typical and atypical strains, a biochemical profile was conducted. Typical and atypical *A. salmonicida* strains were grown under previously described conditions. Growth curves were conducted in triplicate at 15 °C. Briefly, bacteria were grown in 3 mL of TSB media with aeration for 24 h. An inoculum of 300 μL of bacterial culture in the mid-log phase (O.D._600 nm_ ~0.7; ~6.3 × 10^8^ CFU/mL) were added to 30 mL of fresh TSB media and incubated for 48 h at 15 °C with aeration (180 rpm) in an orbital shaker (MaxQ 4000, Thermo Fisher Scientific, Waltham, MA, USA). Bacterial growth was monitored spectrophotometrically until O.D._600 nm_ ~2.0 ± 0.3 using a spectrophotometer (Genesys 10 UV, Thermo Spectronic, Thermo Fisher Scientific, MA, USA).

*A. salmonicida* biochemical profiles were characterized using API ZYM, API20NE, and API20E according to the manufacturer’s instructions (BioMerieux, Marcy-l’Etoile, France). The strips were incubated at 15 °C for 48 h, and the results were analyzed using APIweb (BioMerieux). Additionally, growth under different temperatures (4, 15, 28, and 37 °C) and in different NaCl concentrations at 15 °C (0, 0.5, and 2% NaCl) was evaluated. Motility, catalase, and oxidase activity and the presence of type I fimbria were evaluated using standard methods [7]. The antibiogram was determined for tetracycline (10 μg), oxytetracycline (30 μg), ampicillin (10 μg), sulfamethoxazole (25 μg), chloramphenicol (30 μg), colistin sulfate (10 μg), and oxalinic acid (2 μg) using standard methods [7,30].

#### 2.1.4. Hemolysin Assay

*A. salmonicida* strains were grown as previously described. Bacterial cells were harvested at the mid-log phase, washed three times at 4200× *g* for 10 min at room temperature, and resuspended in 1 mL of PBS [29]. A 5 µL loop was streaked onto TSA plates supplemented with 5% salmon blood or 5% sheep blood and incubated for 48 h at 15 °C and 28 °C.

#### 2.1.5. Siderophores Secretion Assay

*A. salmonicida* strains were grown under previously described conditions. Bacterial cells were harvested at the mid-log phase, washed three times at 4200× *g* for 10 min at room temperature, and resuspended in 1 mL of with PBS. An inoculum of 30 µL was added to 3 mL TSB or TSB supplemented with 100 µM of 2,2-dipyridyl (Sigam-Aldrich, Oakville, ON, Canada). Bacteria were grown at 15 °C with aeration until the mid-log phase. Cells were harvested and washed twice at 4200× *g* for 10 min at room temperature and resuspended in 100 µL of PBS. Five microliters of bacterial suspension was plated onto CAS agar plates [28] and incubated at 15 °C and 28 °C for 48 h.

#### 2.1.6. SDS-PAGE and Western Blot

Protein profiles were determined for typical and atypical *A. salmonicida* strains according to standard protocols [31]. *Escherichia coli* Top10 [32] was used as a control. Bacteria strains were grown in TSB broth 48 h at 15 °C with aeration, except for *E. coli* Top10, which was grown at 37 °C for 18 h. One mL of fresh culture was harvested at 4200× *g* for 5 min at room temperature, washed with PBS, resuspended in 100 µL of 2X SDS-buffer (1M Tris-OH [pH 6.8], glycerol 50%, SDS 10%, bromophenol blue 0.5%, and β-mercaptoethanol 0.5%) [31,33], and boiled for 10 min. A volume of 10 µL of each sample was loaded in 10% acrylamide SDS-PAGE and separated at 120 V for 1:40 h using a Mini-PROTEAN^®^II Cell electrophoresis apparatus (Bio-Rad, Hercules, CA, USA). The gel was stained with a Coomassie blue solution (methanol 50% (*v*/*v*); glacial acetic acid 10% (*v*/*v*); Coomassie blue 0.125% (*w*/*v*); dH_2_O up to 1 L) for 30 min and washed overnight before visualization.

Western blots were performed for the detection of VapA. GroEL, a conserved bacterial chaperon, was used as a positive control. Acrylamide gels and nitrocellulose membranes (0.2 µm, Bio-Rad, Hercules, ON, Canada) were permeabilized in transfer buffer (250 mM Tris-OH; 1.92 M glycine; methanol 20% (*v*/*v*); dH_2_O up to 1 L) for 5 min. Protein transfer was conducted in a semi-dry TRANS-BLOT^®^SD apparatus (Bio-Rad, Hercules, CA, USA) at 20 V for 30 min. Membranes were incubated in blocking buffer (PBS-Tween 0.05% (PBS-T); skim milk 0.5%) overnight, followed by 3 washes with PBS-T for 10 min each. The membranes were incubated with the primary antibody rabbit IgG anti-VapA (1:5000) (Santander’s Lab custom antibody) [26] or rabbit IgG anti-GroEL (Sigma, Oakville, ON, Canada) by shaking (50 rpm) for 1 h at room temperature. Incubation was followed by three washes with PBS-T for 10 min each. The membranes were then incubated with the goat anti-rabbit IgG alkaline-phosphate conjugate (1:5000) (Life Technologies, Thermo Fisher Scientific, Waltham, MA, USA) for 1 h with shaking (50 rpm) at room temperature, followed by three washes with PBS-T for 10 min each. Protein visualization was possible by alkaline phosphatase reaction performed by adding 1 mL of 5-bromo-4-chloro-3-indolyl phosphate (BCIP)-nitro blue tetrazolium (NBT) (Thermo Fisher Scientific, Waltham, MA, USA) to the membrane.

### 2.2. Genomic Analyses

#### 2.2.1. DNA Extraction, Sequencing, Genome Assembly, Annotation, and Mapping

*A. salmonicida* strains were grown under the conditions previously described. Bacterial cells were harvested at the mid-log phase and washed three times with PBS. DNA was extracted using the Wizard DNA extraction High Molecular Weight Kit (Promega, Madison, WI, USA) according to the manufacturer´s instructions. The DNA integrity was evaluated by 0.8% agarose gel electrophoresis and visualized by ethidium bromide staining (50 µg/mL) in an imaging system (iBright 1500). Further, DNA was quantified and tested for purity (260/280 ratio) using spectrophotometry in a Genova-Nano spectrophotometer (Jenway, Staffordshire, UK). Libraries and sequencing were conducted commercially at Genome Quebec, Montréal, ON, Canada) and sequenced using PacBio RS II and Miseq Illumina platforms. The quality of the reads was evaluated using FastQC v.12 (Babraham Institute, Cambridge, UK) [34]. High-quality PacBio sequences were assembled at Genome Quebec using the Hierarchical Genome Assembly Process (HGAP) assembler v.4 [35] with a 30 X coverage (atypical strains) and the Celera Assembler v.3 with 75 X coverage (typical strain). Illumina Mi-Seq sequences were trimmed and assembled with PacBio assembled contigs using CLC Genomics Workbench (CGWB) v.20.0 de novo and genome finishing module tools with default parameters.

Annotation was conducted using the Rapid Annotation Subsystem Technology pipeline (RAST) (http://rast.nmpdr.org/), (last accessed 29 December 2021) [36]. *A. salmonicida* chromosomes and plasmids were submitted to the National Center for Biotechnology Information (NCBI) and re-annotated using the NCBI Prokaryotic Genome Annotation Pipeline (PGAP; 4.10). The whole genome was mapped by using CGView Server (http://cgview.ca/) (last accessed 31 December 2021).

#### 2.2.2. Whole-Genome Alignment and Phylogenetic Analysis

Genomic analyses were conducted using only the completed genomes listed in Table 1. Whole genomes were aligned to calculate the average nucleotide identity (ANI) using the CGWB whole-genome analysis tool with default parameters (Min. initial seed length = 15; Allow mismatches = yes; Min. alignment block = 100; Min. similarity (0.8); Min. length (0.8)). A heat map was computed using the heat map tool with default parameters (Euclidean distance method and complete cluster linkages). Standardized protocols were used to construct a phylogenetic tree [37]. Briefly, the evolutionary history was calculated by the Neighbor-Joining method [38] with a bootstrap consensus of 500 replicates for taxa analysis, and the evolutionary distance was computed using the Jukes–Cantor method [39]. *Pseudomonas putida* KT2440 (NC_002947) chromosome was used as outgroup [40].

The genomes of the virulent strains *A. salmonicida* J410 atypical [41] and *A. salmonicida* J223 typical [26] were selected for additional comparative analyses. Orthologous and homologous genes were compared between strains. Analysis was performed using the Whole Genome Alignment tool in CGWB with default parameters (see above). Dot plots represent homologous regions, orthologous, inversion events, and genome gaps (GGs) within the aligned chromosomes.

#### 2.2.3. Non-Coding RNAs Prediction

The prediction of non-coding RNAs (ncRNAs) was computed for the typical and atypical strains according to a previously described pipeline [23]. Briefly, the prediction was performed using the StructRNAfinder tool [52] for the identification and annotation of ncRNAs based on their secondary structure and covariance models, including Infernal [53], RNAFOLD [54], and Rfam database (Rfam, v14.0) [55], as integrated tools. Predicted ncRNAs were filtered within intergenic regions through BEDTools: intersect v2.3 with default overlap parameters and -v option to report only features with an absence of overlap [56]. Filtered ncRNAs were compared within the four strains and plotted using ggplot2 [57] and jvenn [58]. Analysis was computed for shared and unique ncRNAs for each strain; all copies of a single ncRNA were considered.

#### 2.2.4. Virulence Factors, Insertion Sequences (ISs), and Antibiotic Resistant Gene Analysis

Virulence factors were identified in the chromosome and plasmids using the Virulence Factor DataBase (VFDB) [59,60]. The identification of insertion sequences (ISs) was performed using ISfinder pipeline [61], according to the standardized protocol. Antibiotic resistant-associated genes analysis was performed using the Comprehensive Antibiotic Resistant Database (CARD), sequence analysis was computed by the Resistance Gene Identifier tool (RGI) (https://card.mcmaster.ca/analyze/rgi) (last accessed: 14 December 2021) [62].

### 2.3. Statistical Analysis

All data shown in growth curves graphics were performed in triplicate and evaluated by the mean ± error (SEM). Graphics were performed using GraphPad Prism 7 (GraphPad Software, San Diego, CA, USA).

## 3. Results

### 3.1. A. salmonicida Phenotypic Characteristics, Biochemical, and Enzymatic Profiles

**Figure 1:**
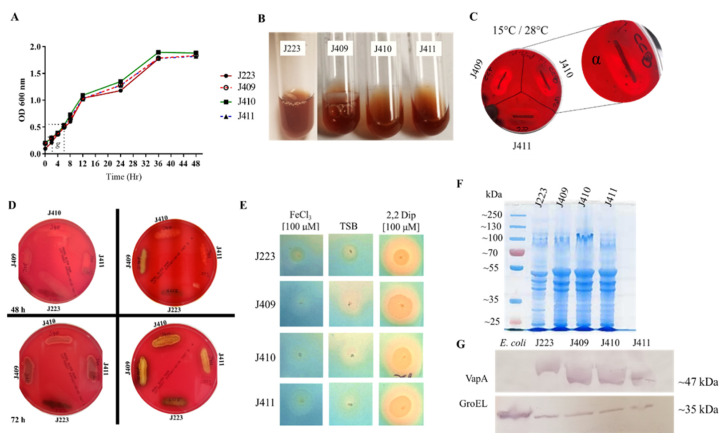
Phenotypic characteristics of atypical and typical *A. salmonicida*. (**A**) Growth curve of *A. salmonicida* J409, J410, and J411 on TSB media at 15 °C; (**B**) *A. salmonicida* melanin-brown pigmentation development on the stationary phase; (**C**) Hemolytic activity evaluated at 15 °C and 28 °C on 5% salmon blood-agar plate for of *A. salmonicida* J409, J410, and J411. The close-up shows α-hemolysin activity; No differences were observed between plates; (**D**) Hemolytic activity evaluated in 5% sheep blood agar plate during 48–72 h at 15 °C and 28 °C; (**E**) Siderophores secretion of *A. salmonicida* strains grown under iron-rich (100 µM FeCl_3_), TSB media, and iron-limited (100 µM 2,2 dipyridyl) conditions, evaluated in CAS-MOPs plates at 15 °C; (**F**) Protein profile evaluated in a 10% SDS-PAGE stained with Coomassie blue; (**G**) VapA protein identification by Western blot; GroEL protein was used as load control, and *E. coli* Top10 and *A. salmonicida* J223 were used as control.

Typical and atypical *A. salmonicida* strains were capable of growing in TSB at different temperatures, with optimal growth at 15 °C (Table 2). The three atypical strains and the typical strain characterized here showed a similar growth rate (*g* = 4 h) at 15 °C and reached the stationary phase in ~36 h (Figure 1A), but they were not capable of growing at 37 °C, indicating a psychotropic growth (Table 2). Additionally, all the strains studied here showed a halophilic phenotype, lack of motility, and lack of type 1 fimbria (Table 2), and brown pigmentation in the late stationary phase (Figure 1B). Hemolytic activity was evaluated in 5% salmon blood (Figure 1C) and in 5% sheep blood agar (Figure 1D) plates. Early α-hemolysin activity was observed in atypical strains at 48 h post-incubation (Figure 1C), which became more prominent at 72 h (Figure 1D). The typical strain J223 presented late α-hemolysin activity compared to the atypical ones, and at 72 h post-incubation, the hemolytic activity became evident at 28 °C (Figure 1D). In addition, the synthesis and secretion of siderophores were observed after 48 h of incubation at 15 °C in all of the *A. salmonicida* strains tested (Figure 1E). These results indicate that the four sequenced strains can synthesize and secrete siderophores and consequently sequester iron from the host during infection (Table 2).

SDS-PAGE profiles and VapA expression analyses were conducted. Atypical tested positive for VapA protein (A-layer) and showed an approximated molecular weight to *A. salmonicida* J223 VapA (Figure 1F,G), indicating that these atypical strains isolated from sablefish belong to the subspecies *salmonicida*. Additionally, these atypical *A. salmonicida* strains showed a positive oxidase and catalase activity, except for the J411 strain, which showed a negative catalase activity (Table 2).

The antibiogram results indicated that all *A. salmonicida* strains studied here were resistant to the *Vibrio* static agent O-129 (Table 2). Additionally, strains J223, J409, and J410 were ampicillin-resistant but susceptible to tetracycline, oxytetracycline, sulfamethoxazole, chloramphenicol, colistin sulfate, and oxolinic acid (Table 2). In contrast, *A. salmonicida* J411 was susceptible to all antibiotics, including ampicillin (Table 2). The enzymatic profile of typical and atypical *A. salmonicida* strains showed minor differences. All these isolates produced alkaline phosphatase, esterase lipase (C_8_), lipase (C_14_), acid phosphatase, and naphthol-AS-BI-phosphohydrolase (Table S1). However, differences in the production of esterase (C_4_), leucine, valine, cysteine arylamidase, β-glucosidase, and N-Acetyl-β-glucosaminidase were noticed between strains (Table S1). Additionally, similarities in biochemical profiles were observed, such as a reduction in nitrates, glucose fermentation, hydrolysis of arginine, esculin, and gelatin and the assimilation of mannose, mannitol, N-acetyl-glucosamine, and malic acid (Tables S2 and S3). Nonetheless, only atypical strains showed trypsin and β-galactosidase activity, acetoin and indole production, hydrolysis of lysine and ornithine, and assimilation of arabinose, potassium gluconate, capric acid, trisodium citrate, and saccharose utilization (Tables S2 and S3), in contrast to typical strain J223, which showed only the synthesis of β-glucosidase, reduction in maltose, mannitol, and amygdaline (Tables S2 and S3).

### 3.2. Genome Sequencing and Annotation

The integrity of *A. salmonicida* high molecular weight (HMW) genomic DNA (gDNA) was evaluated prior to sequencing (Figure S1A,B). Atypical *A. salmonicida* assembled and annotated genomes were submitted to NCBI under the BioProject PRJNA594426 and under the BioSamples SAMN13518349, SAMN13518350, and SAMN1351835 for J409, J410, and J411 strains, respectively (Table S4). One chromosome and one large plasmid were obtained for each strain, with a coverage of 242 X, 277 X, and 108 X for *A. salmonicida* J409, J410 and J411, respectively. The typical *A. salmonicida* J223 genome was submitted to NCBI under BioProject PRJNA310296 and BioSample SAMN04449844 in 2016 as a scaffold and updated as a completed genome in 2020, with a coverage of 160 X (Table S4).

**Figure 2:**
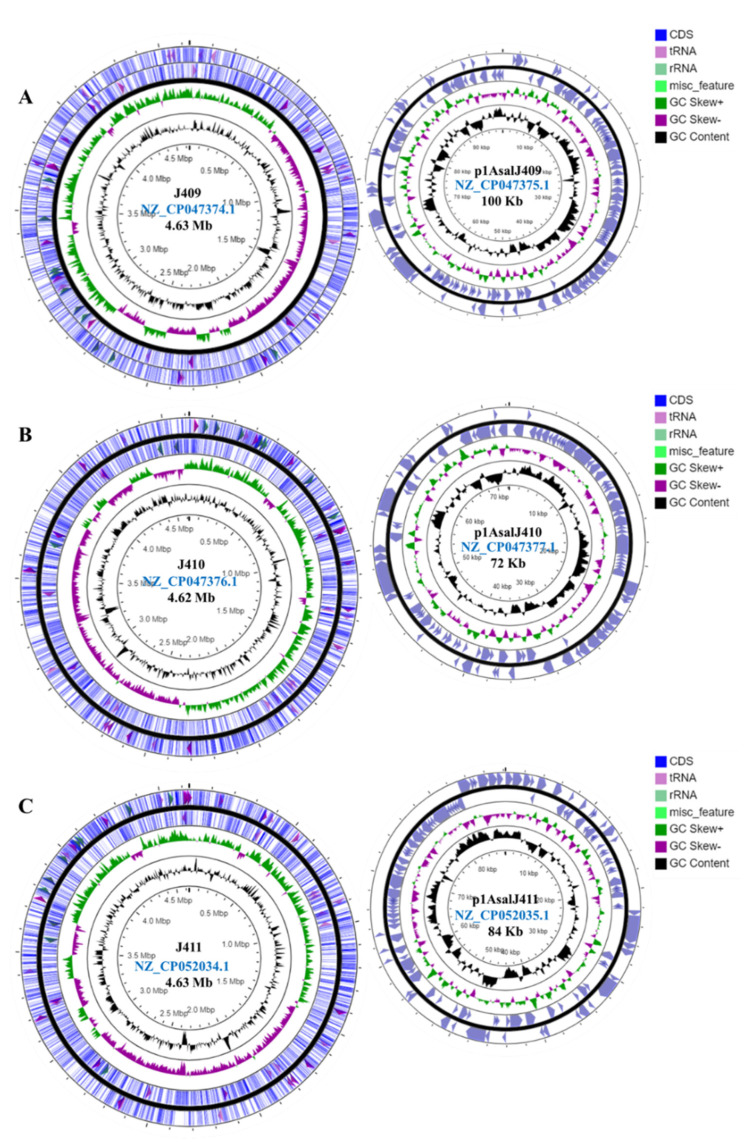
Atypical *A. salmonicida* genome maps. Genome map visualization of *A. salmonicida* genome from J409 (**A**), J410 (**B**), and J411 (**C**). Mapping was performed using the CGViewer pipeline. Maps shown in this figure are not scaled to each other. Left maps represent the chromosome and right map the plasmid. Color bars represent CDS (blue); Color arrows indicate tRNAs (magenta) and rRNA (faint green), GC content (black), GC Skew (+) (forest green), and GC Skew (−)(violet).

Plasmid profiles for the three atypical strains indicated the presence of a large plasmid in each genome (Figure S1). The chromosome sizes of *A. salmonicida* J409, J410, and J411 are very similar, 4.63, 4.63, and 4.63 Mb, respectively (Figure 2A–C). The plasmids present in the genome of atypical *A. salmonicida* strains showed different sizes, ranging between 72 and 100 Kb (Figure 2A–C; Table S4). The G+C content for the three genomes is ~58.6% (Table S5). RAST annotation showed a total of 365, 368, and 366 subsystems (i.e., sets of related functional roles) predicted for J409, J410, and J411 chromosomes, respectively (Table S5).

**Figure 3:**
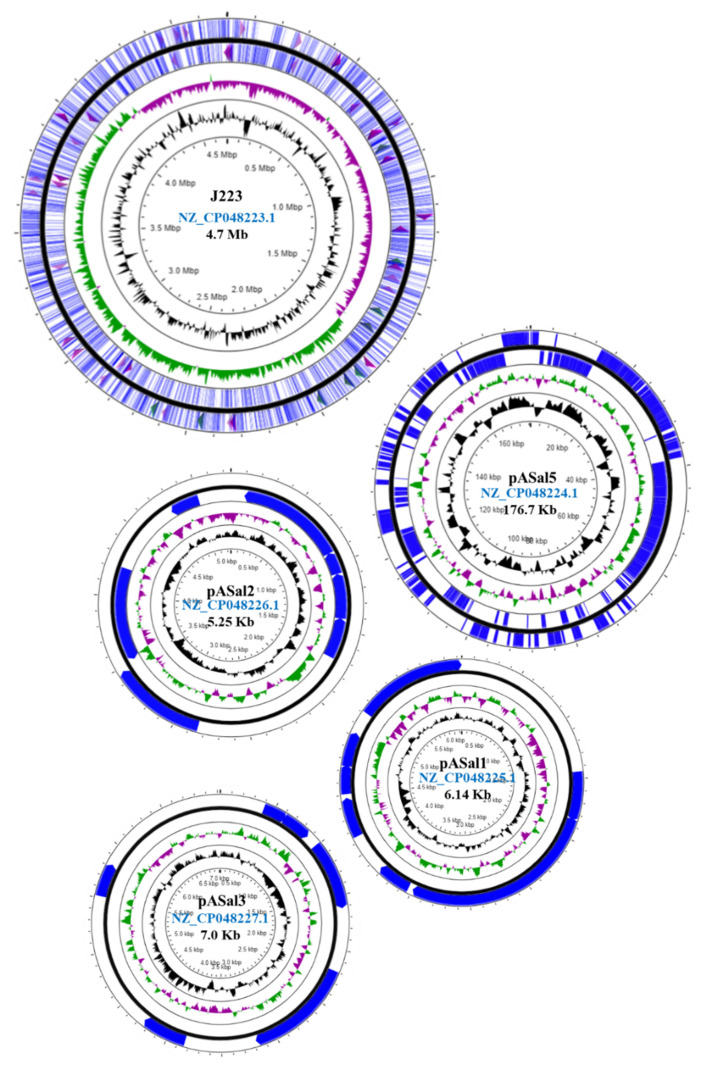
Typical *A. salmonicida* genome map. Genome map visualization of *A. salmonicida* J223. Map located at the upper-left represents the chromosome, and maps located below (central-right) represent the plasmids. Color bars represent CDS (blue); Color arrows tRNAs (magenta), rRNA (faint green), GC content (black), GC Skew (+) (forest green), and GC Skew (−) (violet).

The typical *A. salmonicida* plasmid profile showed the presence of a large plasmid (Figure S1D) and three small plasmids (Figure S1E). The chromosome size of *A. salmonicida* J223 is ~ 4.7 Mb, and the small plasmids pASal1, pASal2, and pASal3 have a molecular size of 6.14, 5.25, and 7.0 Kb, respectively. The large plasmid pASal5 showed a molecular size of 176.7 Kb with a G+C content of 54.8% (Figure 3, Table S4). The RAST annotation showed 344, 1, and 5 subsystems in the chromosome, pASal3, and pASal5, respectively, whereas plasmid pASal1 and pASal2 do not have subsystems features (Table S5).

The Prokaryotic Genome Annotation Pipeline (PGAP) showed a total of 4532 genes, 11 (5S), 10 (16S), and 10 (23S) rRNAs, 116 tRNAs, and 4 ncRNAs for *A. salmonicida* J409 genome; a total of 4546 genes, 11 (5S), 10 (16S), and 10 (23S) rRNAs, 116 tRNAs, and 4 ncRNAs for *A. salmonicida* J410 genome; and a total of 4586 genes, 11 (5S), 10 (16S), and 10 (23S) rRNAs, 121 tRNAs, and 4 ncRNAs for the *A. salmonicida* J411 genome (Table 3). Typical *A. salmonicida* J223 PGAP showed a total of 4626 genes, 10 (5S), 9 (16S), 9 (23S), 116 tRNAs, and 4 ncRNAs (Table 3).

### 3.3. Comparative Analyses of A. salmonicida Chromosome

**Figure 4:**
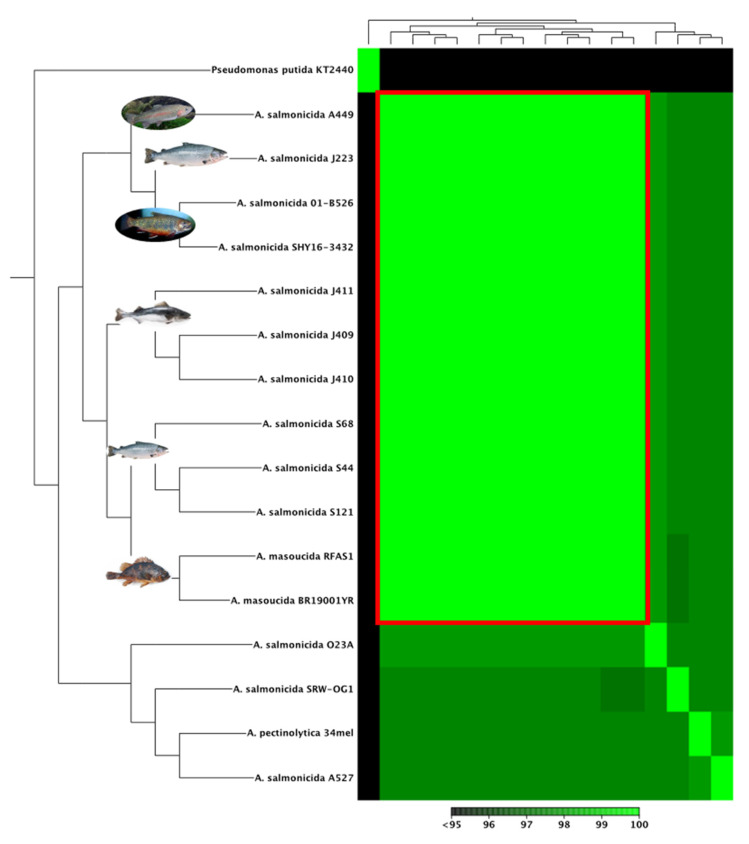
Comparative *Aeromonas* spp. whole-genome analysis. Heat map analysis visualization based on aligned *Aeromonas* spp. chromosomes. Images represent the origin of isolation. Color bar represents the percentage of identity within strains. Thirteen *A. salmonicida* subsp. *salmonicida,* two *A. salmonicida* subsp. *masoucida* RFAS1 and BR19001YR, and *A. salmonicida* subsp. *pectinolytica* 34mel were used for the analysis. *Pseudomonas putida* KT2440 was set up as an outgroup. Analysis was computed using CGWB.

Whole-genome alignment, phylogenetic, and synteny analyses of *A. salmonicida* genomes were performed using completed chromosome sequences (Table 1). High genome identity was observed within the *A. salmonicida* subsp. *salmonicida* analyzed (Figure 4). Specifically, atypical *A. salmonicida* genome identities were 99.50% between strains J409 and J410, 99.33% between strains J409 and J411, and 99.80% between strains J410 and J411 (Figure S2). Typical *A. salmonicida* J223 showed 99.17% identity with *A. salmonicida* strain A449, 98.72% with strain O1-B526 and 98.75% with strain SHY16-3432 (Figure S2).

**Figure 5:**
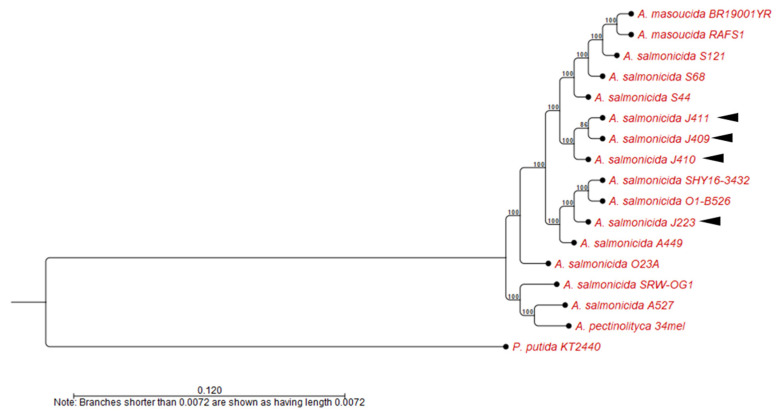
*A. salmonicida* phylogenetic analysis. Phylogenetic history of *A. salmonicida* genomes. Evolutionary history was calculated using neighbor-joining method, with a bootstrap of 500 replicates for taxa analysis. Evolutionary distances were computed by the Jukes–Cantor method. Ambiguous positions were removed with the pairwise deletion option. Analysis was computed using CGWB.

**Figure 6:**
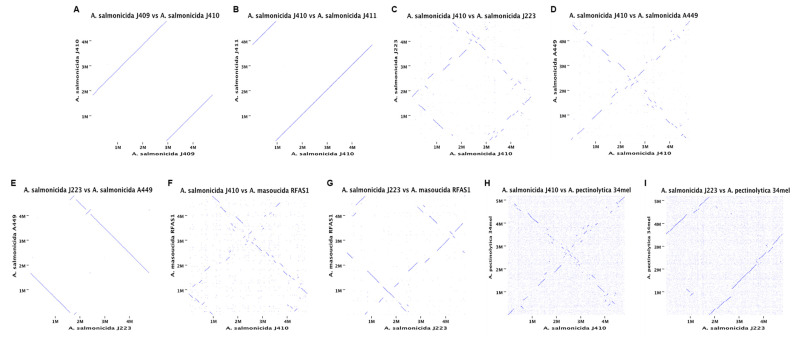
Whole-genome comparison synteny analysis. Genome sequences alignment to identify homology by synteny. (**A**) Comparison between *A. salmonicda* J409 vs. J410; (**B**) Comparison between *A. salmonicda* J410 vs. J411; (**C**) *A. salmonicida* J410 vs. *A. salmonicida* J223; (**D**) *A. salmonicida* J410 vs. *A. salmonicida* A449; (**E**) *A. salmonicida* J223 vs. *A. salmonicida* A449. (**F**) *A. salmonicida* J410 vs. *A. masoucida* RFAS1; (**G**) *A. salmonicida* J223 vs. *A. masoucida* RFAS1; (**H**) *A. salmonicida* J410 vs. *A. peptinolytica* 34mel; *(***I**) *A. salmonicida* J223 vs. *A. peptinolytica* 34mel. Comparative analysis was performed by using CLC Bio.

The phylogenetic analysis showed four clusters with three or more strains. Only *A. salmonicida* subsp. *salmonicida* strains O23A clustered separately. *A. salmonicida* strains J409, J410, and J411 belong to the same cluster (Figure 5), closely related to *A. salmonicida* subsp. *salmonicida* strains S44, S121, and S68 (Figure 5). On the other hand, *A. salmonicida* J223 showed a close relationship to the strains A449, O1-B526, and SYH16-3432 (Figure 5). Interestingly, *A. salmonicida* subsp. *masoucida* RFAS1 and BR19001YR showed high genome identity within *A. salmonicida* subspecies (Figure 4) clustered together with the strains S44, S121, and S68 (Figure 5). The synteny analysis showed that the J409, J410, and J411 chromosomes have high similarity (Figure 6A,B), which agrees with the phylogeny and heat map results (Figure 4 and Figure S2). Genome gaps (GGs) were not observed in atypical chromosomes (Figure 6A,B), but when comparing the genome of atypical strain J410 (selected for the analysis) with typical *A. salmonicida* J223 and A449 GGs, inversion events and orthologous were observed abundantly (Figure 6C,D).

Typical *A. salmonicida* J223 showed high similarity with the A449 strain. However, three inversion events were observed (Figure 6E) that agreed with the percentage of identity previously observed (Figure S2). A low similarity was observed between strains J410 and *A. masoucida* RAFS1 (Figure 6F), and the same results were observed between J223 and RAFS1 (Figure 6G). Shorter homologous regions and an increasing number of inversion events and orthologous were observed in these chromosomal comparisons. While lower similarity was observed for both strains compared with *A. salmonicida* subsp. *peptinolytica* 34mel, the farthest related strain (Figure 6H,I). These results suggested that atypical *A. salmonicida* J409, J410, and J411 share a common ancestor. These *A. salmonicida* isolated from sablefish were closely related to typical *A. salmonicida* S44, S121, and S68 strains. Additionally, these strains were far related from the typical *A. salmonicida* J223, which was closely related to *A. salmonicida* A449 with a high genome similarity. Furthermore, these results also suggested that *A. salmonicida* subsp. *masoucida* strains RFAS1 and BR19001YR might belong to the subsp. *salmonicida*.

### 3.4. Pathogenesis and Environmental Adaptation-Associated Genes in A. salmonicida Genome

Gene distribution was determined for virulence and environmental survival-related genes for atypical (File S1) and typical (File S2) sequenced *A. salmonicida* strains. In the chromosome, genes related to iron homeostasis, ferrous and ferric transport, and regulation mechanisms were identified, including the ferric iron uptake transcriptional regulator (*fur*), *feoB*, and *fhuB* uptake system. A common gene encoding for a siderophore amonabactin TonB-dependent receptor was identified among the four strains, but a siderophore amonabactin export MFS transporter was identified only in atypical strains. Heme transport-associated genes, such as *hutX*, *hutZ*, *ccmA, ccmB, ccmD,* and *ccmF*, were also identified among the four strains (Files S1 and S2). These results agreed with the observed phenotype for iron-uptake and siderophores secretion in the CAS-MOPs assay (Figure 1E).

Metalloproteases-encoding genes, such as *pmbA, tldD*, and *fstH,* and chemotaxis-related genes, such as *cheD*, *cheY*, *cheX*, and *cheV*, were found in all the sequenced strains. However, differences that could impact chemotaxis were observed between strains. The glutamate methyl esterase-encoding gene *cheB* was present in typical J223 and in atypical strains J409 and J410, but it was absent in the J411 strain (File S1). The *cheA* gene that encodes for a cytoplasmic histidine kinase, essential for chemotaxis [63], was present only in the J223 genome in two copies (File S2). Both *cheV* and *cheW*, which play a role in the core of the sensory system of chemoreceptor proteins [63], were identified in the J223 strain in more than a copy; four copies of *cheV* and three copies of *cheW*, respectively (File S2).

Motility-associated genes were identified in all sequenced genomes. Type IV pili-associated genes were identified in the sequenced chromosomes, but some of the genes that required synthesizing a complete type IV pili were not identified in some strains. For instance, *flpL* was absent in J411, and *flpK*, *flpJ*, and *flpD* were absent in J410 (Files S1 and S2). Only one mannose-sensitive hemagglutinin (Msh)-like type IV pilus-encoding gene (*mshL*) was identified in all the atypical strains. In contrast, typical J223 strain harbors the *mshL* gene, and additionally genes such as *mshQ*, *mshP*, *mshD*, *mshL*, *mshK,* and *mshI* were also present (File S2), whereas these are absent in the atypical genomes. Similarly, the *fimV* gene that encodes for a peptidoglycan-binding protein, which promotes the assembly of type IV pilus was found only in the J223 chromosome (File S2).

Atypical strains J409 and J411 and the typical J223 strain harbor the main operon *fliDEFGHIJKLMNOPQR* for flagella synthesis, but essential genes, such *fliH* and *fliK* [64], were truncated by ISs and thus not functional. In contrast, *A. salmonicida* J410 harbors the main flagella operon, but *fliH* and *fliK* are absent. The *fliA* gene, essential for the polar flagella assembly [64], was found only in the atypical strains. Similarly, the *flaE* gene involved in flagellum basal body formation and chemotaxis was found in typical J223 and atypical J410 and J411 strains but is not present in the atypical J409 genome. Intrinsic genes for flagellar assembly, such as *flgLKJIHGFEDCBA*, were identified in a single operon in the J223 chromosome, along with gene copies of *flgK*, *flgHGFE*, *flgCBA,* and *flgN* (File S2). In contrast, only a few flagellar genes were identified in the J410 strain (File S1). Additionally, *pomA*, an ortholog of the MotA motor protein, was identified in all the strains. These results indicated that most of the genes required for motility are present in the sequenced genomes. However, genes required for flagella assembly and functional motility are missing, which correlate with the Mot^—^ phenotype of *A. salmonicida* (Table 2).

Type I fimbria-encoding genes were not identified, which agrees with the negative phenotype Fim^—^.

Secretion systems (SS)-associated genes were identified in the four strains sequenced. All the genes required for the type II secretion system (T2SS) are present in all the sequenced strains. Interestingly, several copies of *gspB*/*exeG* are present in the genome of typical and atypical sequenced strains (Files S1 and S2).

Type III secretion system (T3SS)-associated genes were identified mainly in the large plasmid of typical and atypical strains. However, *aopH* and *aexT* genes that encode for phosphotyrosine phosphatase and ADP-ribosyltransferase toxins, respectively, were found in the chromosome. The atypical strains, J409 and J411, have three copies of *apoH* and a single copy of *aexT* (File S1). In the typical strain J223, the *aopH* gene is not present in the chromosome, and a single chromosomal copy of the *aexT* was identified (File S2).

Type VI secretion system (T6SS)-associated genes, such as *tagO*, *hcp*, *tssA*, and *vgrG,* were identified in the chromosome of all sequenced strains. However, the *tssA* gene that encodes for an essential T6SS core subunit was identified as a pseudogene in the typical J223 strain and in the atypical J409 and J410 strains, in contrast to the J411 strain, which harbored a functional gene sequence (File S1). Interestingly, the *vgrG* gene that encodes for the T6SS spike, an essential unit for functionality [65,66], was identified as a pseudogene in the four strains (File S1 and S2). Structural T6SS genes were mainly found in the typical J223 strain in two operons *tssBCEFG* and *tssJHG* and a single *tssM* gene (File S2). In contrast, atypical strains possess only the *tssM* gene, which encodes for a unit of the T6SS structural complex (File S1).

Genes encoding for hemolysins and toxins were also identified in the chromosome of each sequenced strain, including a thermostable hemolysin III and *ahh1* hemolysin. The hemolysin expression modulating protein (GO993_03655) was identified as a pseudogene only in *A. salmonicida* J411 (File S1). Among toxin-associated genes, an aerolysin family beta-barrel pore-forming toxin (*aerA*) was identified in the four strains, and a type II toxin–antitoxin system VapC was identified only in atypical J411 strain.

Transcriptional regulators, either involved in activation or repression of genes associated with virulence, metabolism, quorum-sensing, cell-division, such as *cysB*, *asnC*, *nhaR*, *hfq*, were found in the chromosome of all sequenced *A. salmonicida* strains. Interestingly, transcriptional regulator genes from the LuxR family were identified in several copies in J409 (2 genes), J410 (2 genes), J411 (4 genes) (Files S1), and the typical J223 strain (4 genes) (File S2), respectively. Additionally, transcriptional regulator-encoding genes from the LysR family were present 47 times in J409, 48 times in J410, 48 times in J411 (File S1), and 45 times in the J223 chromosome (File S2). These results suggest that these transcriptional regulators may play an important role in *A. salmonicida* gene expression associated with quorum sensing.

Antibiotic-resistance genes were only identified at the chromosomal level in the four strains. According to CARD analysis, two antibiotic-resistance genes were identified within the three atypical strains. A resistance-nodulation-cell division (RND) antibiotic efflux pump (*adeF*) that conferred resistance to fluoroquinolone and tetracycline, and an elfamycin-resistant EF-Tu family gene that conferred resistance to pulvomycin (Table S6). Similar results were obtained within the J223 genome, where an elfamycin EF-Tu gene, two *adeF* genes, two beta-lactamase genes TRU-1, and *cphA5* were identified in the J223 chromosome (Table S7). These results agreed with the J223 strain ampicillin-resistant phenotype described by Cipriano in 1991 [26]. We did not identify ampicillin-resistant genes in *A. salmonicida* J409 or J410 from the CARD analysis. However, we manually identified a pseudo-beta-lactamase gene, two subclass B2 metallo-beta-lactamase (*bla*) genes (one pseudogene), and one class D beta-lactamase (*blaOXA*) gene in the atypical strain J409 (Table S6). The atypical strain J410 possesses a beta-lactamase gene, two *bla* genes (one pseudogene), and *blaOXA* (Table S6).

In contrast, the atypical strain J411 possesses a beta-lactamase pseudogene, two pseudo-*bla* genes, and a *blaOXA* gene (Table S6). These results agreed with the previous ampicillin-resistant phenotype observed in strains J409 and J410 but did not agree with the phenotype observed in strain J411 (Table 2). Similar results were found in the typical strain J223, which possesses an *ampC* gene, a pseudo-*blaOXA*, and a CphA family subclass B2 metallo-beta-lactamase-encoding gene (Table S7), consistent with the phenotype and the CARD analyses performed.

### 3.5. Plasmidome of A. salmonicida Sequenced Strains

As previously mentioned, all the atypical *A. salmonicida* strains sequenced in this study harbor one large plasmid (Figure 2A–C; Table S4). In contrast, the highly virulent typical *A. salmonicida* J223 strain harbors four plasmids—one large (>10 Kb) and three small (<10 Kb) plasmids (Figure 3). The typical *A. salmonicida* large plasmid pASal5 (177.0 Kb) has a larger molecular weight and coding sequences (CDS) than the atypical large plasmids (p1AsJ409, 100 Kb; p1AsJ410, 72 Kb; p1AsJ411, 84 Kb; Table S4).

The plasmidome of the typical strain J223 presents genes associated with the type II toxin–antitoxin system. RelE/ParE toxin–antitoxin system family is present in pASal1, pASal3, and pASal5 (File S2). The RelB/DinJ and the VapC family toxin–antitoxin systems are present in pASal1, and pASal5, respectively (File S2). Conversely, atypical strains do not present toxin–antitoxin systems in their plasmid but have them incorporated into the chromosome instead.

**Figure 7:**
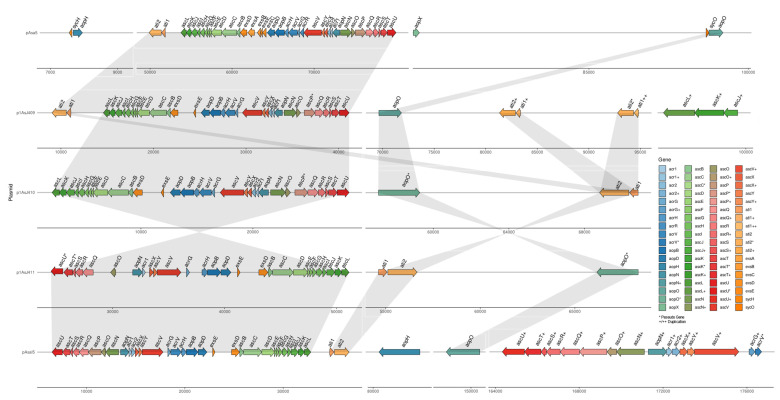
Typical and atypical type III secretion system gene structure comparison. Atypical A. salmonicida virulent plasmids p1AsJ409, p1AsJ410, and pAs1J411 were compared with each other and with the typical A. salmonicida virulent plasmids pAsa5 (A449 strain, top) and pASal5 (J223 strain, bottom). Three main operons were identified among plasmid sequences. Genes are illustrated by arrows. Similar regions were identified between the plasmids, but differences in their position were observed. Pseudogenes are indicated by an asterisk (*), and gene copies are indicated by a plus symbol (+ for a single copy/++ for two copies).

The type III secretion system (T3SS) is one of the main virulence mechanisms of *A. salmonicida* [67]. The structural genes that encode for the T3SS were identified in all sequenced large plasmids from typical and atypical strains and compared to pAsa5 virulent plasmid from typical *A. salmonicida* A449 (Figure 7, Files S1 and S2). However, major differences in T3SS-related genes were observed between large plasmids. For instance, pAsa5 harbors the three main operons that encode for the basal and needle structure, translocon, regulators, and effectors of the T3SS, including transcriptional regulators *exsA*, *exsB*, and *exsC*, effectors *aopH* and *aopX*, and chaperons *sycH* and *sycO* (putative *apoO* chaperone) (Figure 7). We observed that p1AsJ409 harbors a similar set of operon and genes (e.g., *ascBCDEFGHIJKL*, *acrHVG*, *ascVYX*, *ascN*, and *ascOQRSTU*) and the same orientation as observed in the pAsa5 plasmid (Figure 7, File S1). Additionally, p1AsJ409 harbor gene copies related to the T3SS structure (*ascL*, *asc*K, and *ascJ*) and effectors (*ati1* and *ati2*). However, genes such as *ascP* (needle length control) were found as a pseudogene; genes such as *acrR* (chaperon) and specific transcriptional regulators (e.g., *exsA*, *exsB* and *exsC*) are not present in p1AsJ409 (Figure 7, File S1). Similar results were observed in p1AsJ410 that harbors several T3SS-associated genes, sharing a similar orientation and gene context with pAsa5 and p1AsJ409 (Figure 7). However, genes such as *ascP* and *aopO* are also present as pseudogenes. In contrast, p1AsJ411 present several genes associated with the T3SS and a reverse gene context that is similar to pASal5 but different compared to pAsa5, p1AsJ409, and p1AsJ410 (Figure 7). In addition, genes such as *aopO*, *ascT*, and *ascU* were identified as pseudogenes, while genes related to the basal ring and needle structure (*ascP* and *ascN*), needle tip (*acrV*), and effectors (*acr2*) were not identified. These results indicate that the three atypical *A. salmonicida* plasmids sequenced are virulence plasmids that could harbor a functional T3SS and share some similitude between them.

T3SS-encoding genes were identified mainly in the pASal5 large plasmid from the typical strain J223. Significant major differences were observed when compared to the pAsa5 plasmid and the atypical strains’ large plasmids. For instance, pAsal5 harbour genes associated to the T3SS’s basal and needle structure (*ascBCDEFGHIJKL*), needle tip (*acrV*), translocon (*aopD, aopB, acrH*), effectors (*ati1, ati2, aopO, aopH*), and regulation (*aopN, acr1, acr2, acrG, exsE, exsD*). Additionally, gene copies were identified for the basal structure (*ascU*, *ascT*, *ascS*, *ascR, ascQ, ascO, ascN, ascX, ascY, and ascV*), effector (*aopN, acr1*, and *acr2*), and AcrV chaperone (*acrG*) (Figure 7). Also, we identified genes encoding for a phosphotyrosine phosphatase (*aopH*) and a non-functional copy (pseudogene) of *acrV* (Figure 7, File S2). These results indicate that typical *A. salmonicida* J223 possesses a functional T3SS and alternative genes for the regulation and structure of T3SS in the virulence plasmid pAsal5 that are not present in either pAsa5 or atypical plasmids (Figure 7).

### 3.6. A. salmonicida Insertion Sequences (ISs)

**Figure 8:**
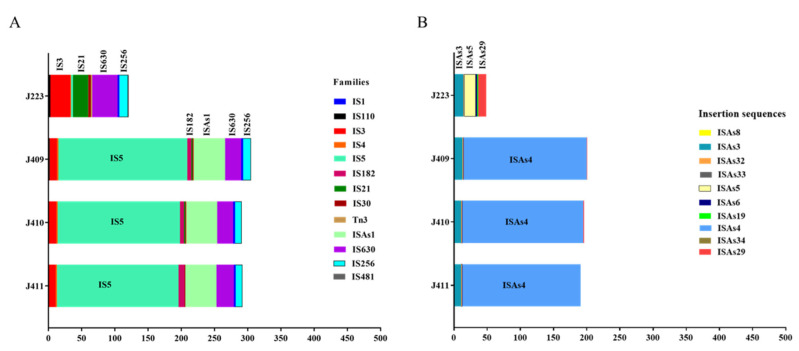
Distribution of insertion sequences’ abundance in typical and atypical *A. salmonicida* genome. (**A**) The graph represents the total IS families identified in the chromosome and plasmids of typical and atypical sequenced strains; all ISs were considerate. (**B**) Representation of the identified ISs and their respective copy numbers as described in Table S8 (last accessed: 9 November 2021). Graph was computed using GraphPad Prims v.9.

Insertion sequences (ISs) play an important role in the *A. salmonicida* genome’s flexibility and pathogenic evolution [5]. Several predicted families of ISs were identified in the chromosome, followed by the larger plasmid of sequenced *A. salmonicida* genomes (Table S8) (last accessed: 9 November 2021). IS families IS3, IS5, IS182, ISAs1, IS630, and IS256 are more abundant in the genome of atypical strains, while IS families IS1, IS110, IS4, IS21, IS30, and Tn3 are the least abundant. On the other hand, the most abundant IS families identified in the typical strain were IS3, IS21, IS630, and IS256, whereas IS1, IS110, IS4, IS481, IS5, IS30, and Tn3 are the least abundant families (Figure 8A).

Among the identified IS families in the atypical strains, the identified ISs present in a high copy number are IS*As*3 (IS256 family) and IS*As*4 (IS5 family) (Figure 8B, Table S8). In contrast, the ISs present in high copy number in the typical strain J223 are IS*As*3 (IS256 family), IS*As*5 (IS3 family), and IS*As*29 (IS21 family) (Figure 8B, Table S8). Clear differences of ISs’ abundances were observed when comparing atypical to typical *A. salmonicida*. Atypical strains present most of the ISs’ copies in the chromosome, whereas the typical strain presents a higher number of ISs’ copies in the pAsal5 plasmid (Table S8). A similar copy number of the IS*As*3 was observed among the four strains. However, two of the major differences are: i) the copy number of the IS*As*5 (17 copies) present in typical strain J223 only and ii) the copy number of IS*As*4, where 174 copies were identified in the chromosome, and 11, 8, and 4 copies were identified in the p1AsJ409, p1J410, and p1J411 plasmid of atypical strains, respectively. Moreover, the typical strain J233 does not presents copies of IS*As*4 (Figure 8B; Table S8).

These results suggest that the main differences between typical and atypical strain genomes are associated with the presence and copy number of specific ISs. Additionally, the high copy number of the IS*As*4 element present in the chromosome suggested that several recombination processes might have already occurred. On the other hand, the typical strain J223 genome presents a reduced copy number of ISs in the chromosome and a higher abundance in the virulent pASal5 plasmid, suggesting a more pristine chromosome with reduced recombination events, which leads to higher chromosomal instability (Figure 6 and Figure 8).

### 3.7. Typical and Atypical A. salmonicida Non-Coding RNAs Repertory

**Figure 9:**
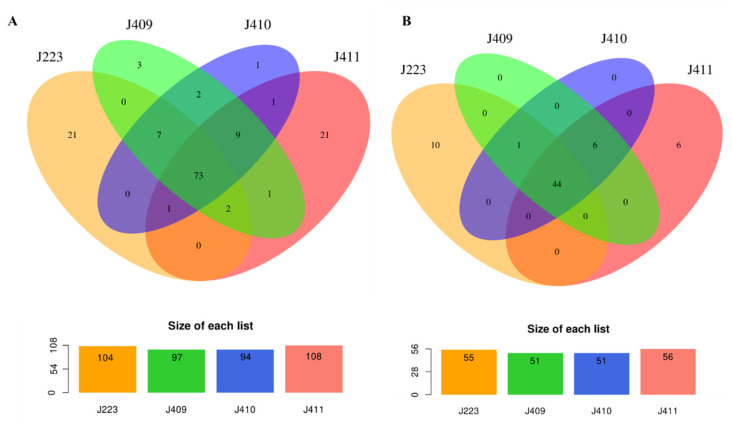
Comparative typical and atypical predicted non-coding RNAs repertory. Venn diagrams show the comparison of ncRNAs repertory identified in typical and atypical strain genomes. (**A**) Graphical representation of total ncRNAs copies identified in typical and atypical *A. salmonicida*, with the list size of number of ncRNAs copies from each strain; (**B**) Graphical representation of single copy ncRNAs, as unique and common shared ncRNAs identified within typical and atypical strains, with the list size of ncRNAs from each strain.

Non-coding RNAs (ncRNAs) were identified within the chromosome and plasmids in the typical and atypical strains (File S3). The analysis showed that the typical and atypical strains possess a similar abundance of ncRNAs. The *A. salmonicida* typical J223 strain harbors 104 ncRNAs copies. *A. salmonicida* atypical strains J409, J410, and J411 have 97, 94, and 108 ncRNAs copies, respectively (Figure 9A). The cluster analysis showed that 73 ncRNA copies are commonly shared among the four strains. The typical J223 strain possesses 21 unique ncRNAs copies and shares seven ncRNAs copies with J409 and J410, two ncRNAs copies with J409 and J411, and one ncRNA copy with J410 and J411 (Figure 9A). On the other hand, atypical strains J409, J410, and J411 have 3, 1, and 21 non-shared ncRNAs copies, respectively. Only two ncRNAs copies are shared between J409 and J410, nine copies are shared between J410 and J411, and one ncRNAs copy is shared between J409 and J411 (Figure 9A).

Several of these ncRNAs are copies of the same ncRNA gene family. When ncRNAs genes are compared, the typical J223 possesses a total of 55 ncRNAs, and atypical strains J409, J410, and J411 possess a total of 51, 51, and 56 ncRNAs, respectively (Figure 9B). Forty-four ncRNAs genes are shared between typical and atypical sequenced strains (File S3, Figure 9B). The typical *A. salmonicida* genome possesses 10 unique ncRNAs and shares one ncRNA with the atypical strains J409 and J410 (File S3, Figure 9B). Atypical strains possess six common ncRNAs (File S3). Similar ncRNAs gene content was observed in J409 and J410. *A. salmonicida* J411 atypical strain presents six unique ncRNAs (Figure 9B; File S3) in contrast to other atypical strains. These results indicate clear differences in the ncRNAs repertory between typical and atypical strains, as well as differences within the atypical strains.

## 4. Discussion

*A. salmonicida* phenotypic identification has been established as Gram-negative, non-motile, non-encapsulated coccobacilli, catalase, and oxidase positive, with optimal growth at 22–25 °C [7,11,68]. In 2015, Gulla et al. described the criteria to determine the *A. salmonicida* subspecies (e.g., *salmonicida*, *masoucida*, *smithia*, *pectinolytica*, *achromogenes*). A series of phenotypic tests are needed to meet these criteria, including a positive phenotype for glucose fermentation, oxidase and catalase activity, and resistance to the vibriostatic agent O/129. Isolates that fulfill all these criteria are considered *A. salmonicida*. Additional tests are required to determine the subspecies. These tests include hemolytic activity, brown pigmentation development, indole production, gelatinase and esculin hydrolase synthesis, assimilation of arabinose and mannitol [11]. Additionally, different characteristics have been attributed to atypical isolates, such as reduced pigmentation, slow growth, ability to grow at temperatures above 30 °C, and, most importantly, their association to an ulcerative disease in cultured and wild fishes [7].

According to these criteria, typical *A. salmonicida* J223 ferments glucose, produces gelatinase, hydrolyzes esculin, assimilates mannitol, catalase, and oxidase, and has hemolytic activity (Table 2, Tables S2 and S3; Figure 1D) but does not produce acid from sucrose or indole, and assimilates arabinose (Tables S2 and S3). This suggests that the J223 strain should be considered typical *A. salmonicida*, but three of these metabolic pathways might be disrupted. In contrast, we observed that atypical strains J409, J410, and J411, isolated from sablefish, were consistent with the atypical description, including a late brown pigmentation development, indole production, glucose fermentation, gelatinase and esculin synthesis, assimilation of arabinose, mannitol and sucrose, catalase and oxidase activity, and hemolytic activity (Figure 1C,D; Table 2, Tables S2 and S3).

The optimal growth temperature is a crucial feature for a typical and atypical phenotype. For instance, the psychotropic phenotype, with optimal growth between 10–22 °C, is characteristic of typical strains [1,68,69]. On the other hand, mesophilic growth is a characteristic of atypical strains, such as *A. salmonicida* Y47, Y567, and Y577 isolated from India, which have optimal growth at 37 °C and an ability to grow at temperatures up to 41 °C [5]. Typical *A. salmonicida* J223 grows optimally between 15 and 28 °C (Table 2), indicating that it is psychotropic. Similar results were observed for the atypical strains J409, J410, and J411, which had optimal growth between 15 and 28 °C (Table 2). Previous studies have described similar results, where strains from the atypical subspecies *smithia*, *achromogenes*, and *masoucida* were identified as psychotropic strains that could not grow at temperatures above 25 °C [70,71]. This suggests that the typical-atypical terminology is irrelevant for *A. salmonicida* taxonomy but could be useful for identifying the isolation source.

In addition, the presence of the A-layer and slow brown pigmentation synthesis has been associated with atypical strains [26]. *A. salmonicida* strains J409, J410, and J411 are very consistent with this previous description, where brown pigmentation was observed during the late stationary phase (Figure 1B). The presence of the VapA protein was evaluated in atypical strains and compared with typical *A. salmonicida* J223 (Figure 1G). A highly conserved VapA sequence between typical strains J223 and A449 and between atypical strains J409, J410, and J411 was observed. However, differences were observed between typical and atypical VapA sequences (Figure S3A) and a similar molecular weight according to predicted 3d structure (Figure S3B,C). A previous study indicated that the geographical origin of the *A. salmonicida* subspecies is related to the VapA sequence or partial sequences [11]. However, atypical *A. salmonicida* subsp. *achromogenes* and *pectinolytica* were excluded from VapA analysis due to a lack of the VapA protein or A-layer in these subspecies [72]. Consequently, the *vapA* gene is not a good marker to organize the *A. salmonicida* species.

Whole-genome analysis has been successfully used for *Aeromonas* classification [47,48,73]. The genome structure of atypical *A. salmonicida* strains J409, J410, and J411 showed a chromosome and a large plasmid (Figure 2). Comparative genome analysis showed that atypical *A. salmonicida* strains J409, J410, and J411 cluster together as isolates from sablefish (Figure 4), with a range of 99.33–99.80% of nucleotide identity (Figure S2). Phylogenetic analysis showed that *A. salmonicida* strains J409, J410, and J411 are closely related and share a common ancestor (Figure 5). They are closely related to typical *A. salmonicida* subsp. *salmonicida* S44, S121, and S68 strains, isolated from Atlantic salmon in the Chinese Pacific Coast [48]. In contrast, the typical *A. salmonicida* J223 genome presents a chromosome, a large plasmid, and three small plasmids (Figure 3). Genome analysis showed that strains isolated from salmonids cluster together with high genome similarity (Figure 4), where the typical *A. salmonicida* strain J223 has 99.21% of nucleotide identity with *A. salmonicida* strain O1-B526 and 99.17% of nucleotide identity with *A. salmonicida* A449 (Figure S2). Similar results were obtained from phylogenetic analysis, where typical *A. salmonicida* J223 is closely related to *A. salmonicida* strain O1-B526 and *A. salmonicida* A449 (Figure 5). These results are consistent with previous studies that established a close relationship between J223 and O1-B526 [74]. These results also confirmed that the *A. salmonicida* strains isolated from sablefish belong to the subsp. *salmonicida*.

Although chromosome sequences share a high identity (Figure S2), the structure showed substantial differences between close-related strains and no structural similitudes with distant strains (Figure 6). This is highlighted by our study for the first time thanks to the analysis of complete closed genomes. Inversion events, genome gaps and orthologous were identified when J410 atypical and J223 typical genomes were compared (Figure 6C), and similar results were observed when comparing J410 and A449 typical strains (Figure 6D). However, high genome identity was observed when comparing typical strains J223 and A449 (Figure S2), in which only three inversion events were observed (Figure 6E). These results are consistent with a previous study in *A. salmonicida* subspecies, where different genome organizations (rearrangements, inversions, genome gaps, etc.) were described when comparing subspecies, such as *masoucida*, *peptinolytica*, *achromogenes*, and *smithia*, with subsp. *salmonicida* [5]. These results revealed that the *A. salmonicida* chromosome is highly plastic, which is likely driven in part by ISs. Additionally, this suggests that typical strains harbor more active ISs than atypical strains, suggesting that atypical strains could have evolved from a recent endogenous mutagenesis event.

Additionally, the number of plasmids harbored by typical strains are larger than atypical strains. The size of the biggest plasmid is greater in typical strains compared to atypical strains. These differences in the plasmidome content between typical and atypical strains were previously noted in J223 and A449, S44, S121, S68, and O1-B526 strains [17]. This has a direct relation with the host origin of the isolate and virulence [42,44], where typical strains are usually salmonid isolates that exhibit an acute virulence but capable of infecting several hosts (Table 1).

Similitudes and differences found in virulence factors might play an important role in typical and atypical strain virulence. For instance, iron-homeostasis, ferrous, and ferric transport-associated genes, regulated by the ferric uptake regulator (Fur) protein, are considered essential for the pathogenesis and survival of *A. salmonicida* [75]. The sequenced typical and atypical strains harbor several iron-related genes (Files S1 and S2), which agreed with the induction of siderophore synthesis under iron-limited conditions (Figure 1E), indicating their capability to secure iron while infecting a host.

Hemolytic activity is also critical for the pathogenesis and virulence of marine pathogens. Typical and atypical strains present a hemolysin III protein, a thermostable hemolysin, and a hemolysin AHH1 (*ahh1*) (Files S1 and S2). AHH1 hemolysin has been previously reported in *A. hydrophila* and as a homologous gene of hemolysin ASH4 in *A. salmonicida* [76]. This agreed with our phenotypic observations, where typical and atypical strains showed α-hemolysin activity (Figure 1D). However, atypical strains showed earlier hemolysin activity at 15 and 28 °C at 48 h of incubation (Figure 1C,D), whereas the typical J223 strain showed hemolysin activity at 28 °C after 72 h of incubation (Figure 1D). These results suggest that the typical J223 strain possesses delayed and thermo-inducible hemolysin activity, but its regulatory mechanism requires further study.

Differences found in the flagella secretion system-associated genes might play an important role in *A. salmonicida* virulence. For instance, most of the genes encoding for flagellar structural proteins were identified in the typical and atypical strains (File S1 and S2). However, genes, such as *fliH* and *fliK*, associated with export/assembly and the hook length, respectively [64], are truncated genes and absent in the J410 genome. Flagella-assembly-associated genes were only identified in the typical J223 genome. The polar flagella and lateral flagella have been previously described and identified across bacteria, among them *E. coli* and *Salmonella* Typhimurium as a model of study [77], including *A. hydrophila* [64,78]. Still very little is known about this system in *A. salmonicida*. These results indicate that the sequenced *A. salmonicida* strains cannot assemble a functional flagellum, which agrees with its Mot^—^ phenotype. However, the flagellar secretion system seems to be functional and is used to induce an inflammatory host response as well as increase attachment to the host cell [79,80,81].

T3SS has been described as the main virulence mechanism of *A. salmonicida*, responsible for the secretion and translocation of several toxins and effector proteins into the host cell, impacting their integrity, modulating phagocytosis, and suppressing the host immune response [67,82,83,84]. T3SS genes that encode for the needle apparatus, regulatory proteins, and effectors have been previously described and identified in the virulence plasmid pAsa5 in the *A. salmonicida* A449 strain (Figure 7) [12]. Here, T3SS genes were mainly identified in the large plasmid of each atypical strain (Figure 7, File S1) and in pASal5 of the typical J223 strain (Figure 7, File S2). Differences were observed when comparing atypical strains T3SS-encoding genes. For instances, effector-encoding genes for phosphotyrosine phosphatase (*aopH*) and ADP-ribosylating toxin (*aexT*) were identified in J409 and J411 chromosomes. Three copies of *aopH* are present in the chromosomes of J409 and J411. However, in the J410 strain, the *aopH* and *aexT* genes are absent (File S1). T3SS transcriptional regulatory genes, such as *exsE* and *exsD*, were identified in the three plasmids, but transcriptional regulators, such as *exsA*, *exsB*, and *exsC*, were not identified as compared to the pAsa5 plasmid [12,83]. Few gene copies encoding for the inner membrane ring (*ascJ*) and cytoplasmic structural portion (*ascK* and *ascL*) and effector genes (*ati1*) were observed in the p1AsJ409 plasmid (Figure 7, File S1). Several pseudogenes were identified within operons as monocistronic genes, most of which were present in p1AsJ411 (e.g., *ascP*, *ascU*, *ascT*, *aopO*) (Figure 7, File S1). Furthermore, in the p1AsJ411 plasmid, we did not identify *acrR*, *acrV aopH ascN,* and *ascP*, which are essential genes for the C-ring and needle structure [82,83]. Additionally, p1AsJ411 presents three inversions when compared to p1AsJ409 and p1AsJ410, and these events agreed with the location of the pseudo-transposases and pseudogenes observed in the plasmids, indicating previous recombination events (Figure 7). These in silico analyses indicated that the three atypical strains harbor a virulent plasmid that carries genes for T3SS. However, the functionality of the T3SS in these plasmids could not be optimal and affects virulence. Further analyses are required to determine their effect on virulence.

In contrast, the typical strain J223 harbors a complete T3SS in the virulent pASal5 plasmid, except for the chromosomal toxin-encoding gene *aexT* (File S2). Additionally, gene copies related to structural proteins and effectors [83,85], such as *aopN* and *acr2* genes (File S2), are orthologous effectors previously described in *Yersinia ruckei* [85]. The presence of *aopN* and *acr2* genes as additional copies indicates that the typical strain J223 is likely to be highly virulent.

These differences observed at the genomic level in terms of virulence factors agree with previous virulence studies. For instance, *A. salmonicida* typical J223 causes an acute infection in rainbow trout (*Oncorhynchus mykiss*) [26], Atlantic salmon [86], and lumpfish [87]. The intra-peritoneal (i.p.) LD_50_ was calculated as 1 × 10^2^ CFU/dose in all fish species tested, and a 1 × 10^3^ CFU/dose caused 100% mortality in 15 days. Mortality in lumpfish infected by bath with the typical *A. salmonicida* J223 strain (3 × 10^6^ CFU/mL) reached 100% in 15 days (data not shown). These data indicate that *A. salmonicida* typical J223 is a host generalist, highly pathogenic strain that causes acute furunculosis. In contrast, the atypical J410 has an LD_50_ dose of ~3 × 10^5^ CFU/dose, which indicates a more chronic infection [41]. Additionally, we believe that the plasmidome could play a critical role in J223 pathogenesis; although it harbors three small cryptic plasmids that do not carry virulence genes, these plasmids could favor and support bacterial metabolic pathways during the infection process, conferring an advantage [17].

The increasing genomic diversity, host range, and environmental adaptation of *A. salmonicida* has likely been driven in part by insertion sequences (ISs) [5,13]. Previous studies described that ISs mediated the loss of virulence of *A. salmonicida* due to endogenous mutagenesis events [15,88]. The events described involve gene inactivation and plasmid rearrangements [15] due to a large number of ISs contained in the genome of *A. salmonicida* [12]. Three major events have been previously described, including: (*i*) loss of the external A-layer protein array by the disruption of the *vapA* gene as result of the spontaneous transposition of IS*As*1 and IS*As*2 [16]; (*ii*) loss of the T3SS locus; (*iii*) loss of the T3SS locus plus 40-Kb upstream region [13,88]. For instance, typical *A. salmonicida* J223 has been described as a psychotropic strain, but it is able to growth at 28 °C (Table 2). Its growth at 28 °C caused severe phenotype modifications in J223, including the loss of the A-layer, which is revealed by white colonies on the Congo red-TSA plates incubated at 28 °C (Figure S2D). This event has been previously described as a result of growth under stressful conditions and ISs’ activity [10,15,88]. This suggests that typical *A. salmonicida* J223 presents thermal inducible ISs. This event represents the genomic flexibility of *A. salmonicida*, which has a direct impact on *A. salmonicida* classification. Nonetheless, this event was not observed within atypical strains J409, J410, and J411 grown at 28 °C, suggesting that recombination processes might have occurred previously, and their chromosome could be more stable.

The loss of the T3SS locus in *A. salmonicida* has been associated with the presence of the mobile genetic element IS*As*11 (also known as IS*As*3), a member of the IS256 family located in the pAsa5 plasmid [12]. Additionally, new ISs have been characterized and described in the *A. salmonicida* genome, such as IS*As*5, as well as IS*As*1 and IS*As*2, which are also involved in gene inactivation and plasmids rearrangement [13]. Atypical *A*. *salmonicida* strains present between 9 copies of IS*As*3 (IS256 family) in the chromosome and 1–3 copies in the plasmid (Table S8). Moreover, they present a single copy of IS*As*32 and IS*As*6 (IS3 family) in the chromosome, and multiple copies of IS*As*4 (IS5 family) in the chromosome and between 4 and 11 copies in their plasmids (Figure 8B, Table S8). The low number of copies of the thermo-inducible IS*As*3 and the absence of IS*As*5 in their plasmids suggest a greater stability of the A-layer after thermal stress at 28 °C. On the other hand, IS5 family, such as IS*As*4, do not have the associated events previously described. However, the presence of multiple copies distributed within the genome indicates that they might be responsible for multiple recombination events that drive the loss of transcriptional regulatory, essential structural T3SS genes and, consequently, pseudogenes in the atypical *A. salmonicida* genomes that might help to understand their host tropism, as previously hypothesized [89].

In contrast, typical *A. salmonicida* J223 presents a total of 13 copies of IS*As*3 identified within the chromosome and the pASal5 plasmid. It additionally presents a single copy of IS*As*32 and IS*As*33, two copies of IS*As*6, and several copies of IS*As*5 in the chromosome, and two copies of IS*As*5 in the pASal5 plasmid (Figure 8, Table S8). Mobile elements IS*As*11 (or IS*As*3) and IS*As*5 have been associated with endogenous mutagenesis at temperatures >25 °C [13,88]. In this scenario, additional copies of IS*As*5 identified in the J223 pASal5 plasmid might be responsible for the loss of the VapA protein and white colony phenotype observed in typical strain J223 after heat shock at 28 °C (Figure S3D). Additionally, additional copies of IS*As*3 could trigger the loss of T3SS in J223, but both hypotheses require further study. These results indicate that typical strain J223 has several active ISs and possesses a more prime genome that does not pass through several recombination processes. This suggests that mobile genetic elements from the IS3 family could trigger several endogenous mutagenesis processes, causing chromosomal instability, and could be responsible for the transition from psychrotroph to mesophilic phenotype or vice versa, as previously described [5].

Non-coding RNAs also play an important role in bacterial virulence and modulate gene expression [90,91]. To improve the *A. salmonicida* genome annotation, we predicted the ncRNAs repertory in the typical and atypical genomes based on their secondary structure [52], providing a useful annotation for expression and functional studies (Supplementary File S3). We identified about 55 shared ncRNAs, whereas 10 unique ncRNAs were identified in typical J223 and 6 unique ncRNAs identified in atypical strain J411. Among these shared ncRNAs, we identified well-characterized ncRNAs, including RyhB, an ncRNA related to iron homeostasis [92,93], and CsrB and CsrC, ncRNAs associated with carbon source storage, motility, and biofilm formation [94,95].

Among the unique ncRNAs in the typical *A. salmonicida* J223, we identified a betaproteobacteria toxic small RNA (tsRNA). tsRNA was previously described in *Burkholderia cenocepacia* and *Herbaspirillum seropedicae*, and the evidence suggests that it could regulate gene expression by binding to the ribosome-binding sequence of the mRNA [96]. Other ncRNAs seem to be related to plasmid replication, such as RNAI (RF00106), an antisense repressor that regulates plasmid replication in ColE1 plasmids [97]. This suggests that RNAI could play a role in the plasmid copy number, allowing *A. salmonicida* J223 to harbor five plasmids. Furthermore, we identified a ncRNA associated with virulence in typical J223 strains but not in atypical strains. For instance, the sRNA-Xcc1 [98] has 15 copies in *A.* *salmonicida* J223 genome, but it is absent in the atypical strains. This suggests that sRNA-Xcc1 could be favored typical *A. salmonicida* J223 virulence; however, this requires further studies. Differences in ncRNAs’ repertory between typical and atypical strains could be related to the mechanism of regulation for metabolic pathways, gene transcription, and post-transcriptional modulation that ultimately impacts virulence.

## 5. Conclusions

This study conducted a comparative genomics analysis of *A. salmonicida* using the whole genomes of three atypical strains isolated from sablefish from the same host and geographic region and one typical strain isolated from Atlantic salmon. These strains showed a high genome identity with each other, closely related to typical *A. salmonicida* strains. The typical strain *A. salmonicida* J223 isolated from Atlantic salmon in 1999 showed a close relationship to the typical European strain A449 with a high genome identity. Genomic differences, such as inversion events, orthologous, genome gaps (GGs), and plasmids, were observed when comparing atypical with typical genomes. Major differences between typical and atypical strains are related to the presence or absence of virulence factors (i.e., transcriptional regulators and structural T3SS genes, effectors, and toxins) and the abundance of insertion sequences (ISs). A high abundance of thermo-inducible active IS*As*3 and IS*As*5 in *A. salmonicida* J223 indicates high genome plasticity related to its high virulence and its ability to infect a broader range of fishes. The abundance of IS*As*4 in atypical *A. salmonicida* suggests that several endogenous mutagenesis events could have already occurred and might contribute to the atypical strains host adaptation. Our study reveals that whole-genome analysis does not agree with the traditional typical and atypical phenotypic classifications. *A. salmonicida* subspecies, plasmid content, and ISs’ repertoire seem to be driving *A. salmonicida* evolution. Comparative genomic analysis based on completed genomes revealed that the subspecies classification is more of a reflection of the ecological niche occupied by bacteria than their divergences at the genomic level except for their accessory genome.

## Figures and Tables

**Table 1:** Geographic origin and host species of *A. salmonicida* isolates.

*A. salmonicida* Subspecies	Geographic Origin, Host Specie	Accession Number	N° of Plasmids	References
*salmonicida* A449	France/ *Salmo gairdneri*	CP000644-46	5	[12,42,43]
*salmonicida* J223 (Wild-type 3.173)	Pittsford, VT, USA, 1999/ *Salmo salar*	CP048223-27	4	[26]/This study
*salmonicida* J409	Canada/ *Anoplopoma fimbria*	CP047374-75	1	This study
*salmonicida* J410	Canada/ *Anoplopoma fimbria*	CP047376-77	1	This study
*salmonicida* J411	Canada/ *Anoplopoma fimbria*	CP052034-35	1	This study
*salmonicida* O1-B526	Canada/ *Salvelinus fontinalis*	CP027000	6	[44,45]
*salmonicida* SHY16-3432	Canada/ *Salvelinus fontinalis*	CP038102-105	7	[46]
pectinolytica 34mel	Argentina/ environmental sample	CP022426	-	[47]
*salmonicida* S44	China/ *Salmo salar*	CP022176-81	5	-
*salmonicida* S121	China/ *Salmo salar*	CP022170-75	5	-
*salmonicida* S68	China/ *Salmo sala*r	CP022182-86	4	-
*masoucida* BR19001YR	South Korea/ *Sebastes schlegeli*	CP060030-33	3	-
*salmonicida* SRW-OG1	China/ *Epinephelus coioiaes*	CP051883	-	-
*salmonicida* A527	India/*Macrobrachium rosenbergii*	CP022550	-	[48]
*salmonicida* O23A	Poland/ environmental sample	CP021654-58	4	[49]
*masoucida* RAFS1	South Korea/ *Sebastes schlegelii*	CP017143-45	2	[50]
*Pseudomonas putida* KT2440	France/ Environmental sample	NC_002947	-	[51]

**Table 2:** Phenotypic characteristics of *A. salmonicida* strains J409, J410, J411, and J223. Except where indicated, all the tests were performed at 15 °C.

Characteristic	J409	J410	J411	J223
Grown in TSB at:				
4 °C	+	+	+	+
15 °C	+	+	+	+
28 °C	+	+	+	+
37 °C	-	-	-	-
LB NaCl 0%	-	-	-	-
LB NaCl 0.5%	+	+	+	+
LB NaCl 2%	+	+	+	+
Motility	-	-	-	-
Fimbria type I	-	-	-	-
Siderophores synthesis	+	+	+	+
Hemolytic activity	+	+	+	+
Catalase	+	+	-	+
Oxidase	+	+	+	+
Antibiogram	mm	mm	mm	mm
Tetracycline (10 µg)	38 (Susceptible)	36 (Susceptible)	16 (Susceptible)	31 (Susceptible)
Oxytetracycline (30 µg)	42 (Susceptible)	40 (Susceptible)	40 (Susceptible)	32 (Susceptible)
Ampicillin (10 µg)	0 (Resistant)	0 (Resistant)	16 (Susceptible)	0 (Resistant)
Sulfamethoxazole (25 µg)	24 (Susceptible)	26 (Susceptible)	39 (Susceptible)	24 (Susceptible)
Chloramphenicol (30 µg)	40 (Susceptible)	36 (Susceptible)	44 (Susceptible)	13 (Susceptible)
Colistin sulphate (10 µg)	12 (Susceptible)	18 (Susceptible)	21 (Susceptible)	16 (Susceptible)
Vibriostatic O-129 (150 µg)	0 (Resistant)	0 (Resistant)	0 (Resistant)	0 (Resistant)
Oxolinic acid (2 µg)	36 (Susceptible)	42 (Susceptible)	18 (Susceptible)	34 (Susceptible)

**Table 3:** NCBI Prokaryotic Genome Annotation Pipeline Summary.

Attribute	Data Provided			
Annotation Pipeline	NCBI prokaryotic Genome Annotation pipeline			
Annotation Method	Best placed reference protein set; GeneMarks v 4.10			
*A. salmonicida* genome	J409	J410	J411	J223
Genes (total)	4532	4546	4586	4.626
CDSs (total)	4381	4395	4430	4.478
Genes (coding)	3948	3985	3456	4.207
Genes (RNA)	151	151	156	148
rRNAs	11, 10, 10 (5S, 16S, 23S)	11, 10, 10 (5S, 16S, 23S)	11, 10, 10 (5S, 16S, 23S)	10, 9, 9 (5S, 16S, 23S)
Complete rRNAs	11, 10, 10 (5S, 16S, 23S)	11, 10, 10 (5S, 16S, 23S)	11, 10, 10 (5S, 16S, 23S)	10, 9, 9 (5S, 16S, 23S)
tRNAs	116	116	121	116
ncRNAs	4	4	4	4
Pseudogenes (total)	433	410	974	271
Pseudogenes (ambiguous residues)	0 of 433	0 of 410	0 of 974	1 of 271
Pseudogenes (frameshifted)	182 of 433	162 of 410	728 of 974	157 of 271
Pseudogenes (incomplete)	250 of 433	243 of 410	263 of 974	90 of 271
Pseudogenes (internal stop)	40 of 433	40 of 410	45 of 974	47 of 271
Pseudogenes (multiple problems)	37 of 433	33 of 410	59 of 974	22 of 271

## Supplementary Materials

The following supporting information can be downloaded at: https://www.mdpi.com/article/10.3390/microorganisms10010189/s1, Figure S1. *A. salmonicida* high molecular weight (HMW) and plasmids; Figure S2. Average nucleotide identity (ANI) table of whole genome alignment; Figure S3. *A. salmonicida* A-layer (VapA) sequence and structure analysis; Table S1. Enzymatic profile of *A. salmonicida* using the API ZYM system; Table S2. Enzymatic profile of *A. salmonicida* using API20NE; Table S3. Enzymatic profile of A. salmonicida using the API 20E; Table S4. A. salmonicida genomes used in this study; Table S5. Summary of the RAST annotation genome results; Table S6. Antibiotic resistance gene identification in atypical A. salmonicida strains; Table S7. Antibiotic resistance gene identification in typical A. salmonicida; Table S8. Insertion sequences abundance in typical and atypical A. salmonicida genomes; File S1. Virulence factors list of atypical A. salmonicida strains; File S2. Virulence factors list of typical A. salmonicida strain. File S3. List of predicted nc-RNAs in typical and atypical *A. salmonicida* strains.

## Abbreviations

°C	Degree centigrade
µL	Microliter
CFU	Colony forming unit
h	Hour
Kb	Kilo base pair
L	Liter
Mb	Mega base pair
mg	Milligram
min	Minute
mL	Milliliter
OD	Optical density
PBS	Phosphate buffered saline
TSB	Tryptic Soy Broth media
TSA	Tryptic Soy Agar
SEM	Standard error of the mean
Subsp.	Subspecies
ISs	Insertion sequences
GGs	genomes gaps
