# Supplementary material for: Comparative Genomics of Typical and Atypical Aeromonas salmonicida Complete Genomes Revealed New Insights into Pathogenesis Evolution
Source: Microorganisms. 2022 Jan 15;10(1):189. doi: 10.3390/microorganisms10010189 (PMC8780938; doi:10.3390/microorganisms10010189)
Supplement: Supplementary file 1 [file microorganisms-10-00189-s001.zip › microorganisms-1512247 supplementary/Supplementary Figures.pdf]

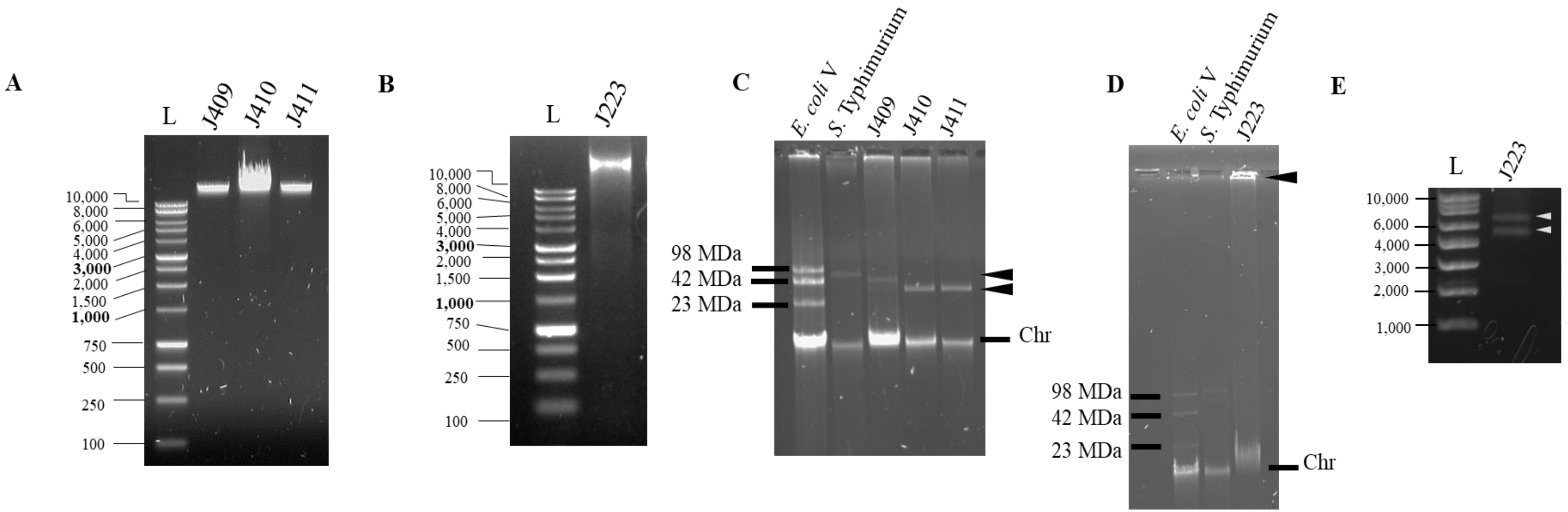

Figure S1. *A. salmonicida* high molecular weight (HMW) and plasmids. **A.** gDNA integrity evaluation by electrophoresis using agarose gel 1% pre-sequencing atypical *A. salmonicida*; **B.** Plasmid DNA extraction by Kado & Liu method for atypical *A. salmonicida.* **C.** gDNA integrity evaluation by electrophoresis using agarose gel 1% pre-sequencing typical *A. salmonicida*. **D.** Plasmid DNA extraction by Kado & Liu method for typical *A. salmonicida.* **E.** Plasmid DNA extraction by mini prep columns method for typical *A. salmonicida.* A supercoiled DNA ladder (Invitrogen, USA) was used for plasmid extraction. Chr = chromosomal DNA.

		1	2	3	4	5	6	7	8	9	10	11	12	13	14	15	16	17
P. putida KT2440	1		0.00	0.00	0.00	0.00	0.00	0.00	0.00	0.00	0.00	0.00	0.00	0.00	0.00	0.00	0.00	0.00
A. pectinolityca 34mel	2	0.12		97.12	97.12	97.12	97.12	97.12	97.12	97.13	97.12	97.12	97.12	97.51	97.19	97.11	97.11	97.41
A. salmonicida J409	3	0.13	81.60		100.00	100.00	99.80	99.81	99.80	99.81	99.85	99.85	99.85	97.12	97.65	99.84	99.84	97.09
A. salmonicida J410	4	0.13	81.88	99.50		100.00	99.80	99.80	99.80	99.80	99.85	99.85	99.85	97.13	97.64	99.84	99.84	97.09
A. salmonicida J411	5	0.13	81.73	99.33	99.80		99.80	99.80	99.80	99.80	99.85	99.85	99.85	97.12	97.64	99.84	99.84	97.08
A. salmonicida A449	6	0.13	82.82	90.46	90.74	90.62		99.98	99.98	99.97	99.81	99.81	99.81	97.15	97.65	99.80	99.80	97.10
A. salmonicida J223	7	0.13	82.79	90.47	90.76	90.63	99.17		99.99	99.99	99.81	99.81	99.81	97.16	97.65	99.80	99.80	97.11
A. salmonicida O1-B526	8	0.13	82.48	90.16	90.44	90.32	98.72	99.21		99.99	99.80	99.80	99.80	97.16	97.64	99.80	99.80	97.11
A. salmonicida SHY16-3432	9	0.13	82.41	90.10	90.38	90.26	98.75	99.25	99.88		99.82	99.81	99.82	97.15	97.63	99.81	99.81	97.11
A. salmonicida S44	10	0.13	82.45	91.48	91.82	91.64	92.24	92.22	91.80	91.83		100.00	100.00	97.13	97.64	99.99	99.99	97.08
A. salmonicida S121	11	0.13	82.38	91.40	91.74	91.56	92.21	92.20	91.77	91.81	99.87		100.00	97.13	97.64	99.99	99.99	97.08
A. salmonicida S68	12	0.13	82.32	91.33	91.67	91.49	92.15	92.13	91.70	91.74	99.80	99.77		97.14	97.64	99.99	99.99	97.08
A. salmonicida A527	13	0.12	84.92	84.21	84.50	84.37	85.60	85.57	85.26	85.23	85.23	85.23	85.13		97.18	97.12	97.11	97.41
A. salmonicida O23A	14	0.13	84.72	85.10	85.37	85.20	87.20	87.24	86.91	86.93	86.76	86.68	86.62	86.34		97.63	97.62	97.18
A. masoucida RAFS1	15	0.15	81.57	90.45	90.79	90.61	91.30	91.28	90.86	90.90	98.39	98.36	98.29	84.53	85.81		99.99	97.04
A. masoucida BR19001YR	16	0.13	81.55	90.43	90.77	90.61	91.34	91.32	90.90	90.93	98.39	98.36	98.29	84.50	85.81	99.69		97.04
A. salmonicida SRW-OG1	17	0.16	85.99	85.69	85.96	85.83	87.07	87.06	86.71	86.71	86.90	86.82	86.76	87.94	88.78	86.22	86.20	

Figure S2. Average nucleotide identity (ANI) table of whole genome alignment. Twelve *A. salmonicida* subsp. *salmonicida,* two *A. salmonicida* subsp. *masoucida* RFAS1 and BR19001YR, and *A. salmonicida* subsp. *pectinolytica* 34mel were used for the analysis. *Pseudomonas putida* KT2440 was set up as an outgroup. Analysis was computed by using CGWB.

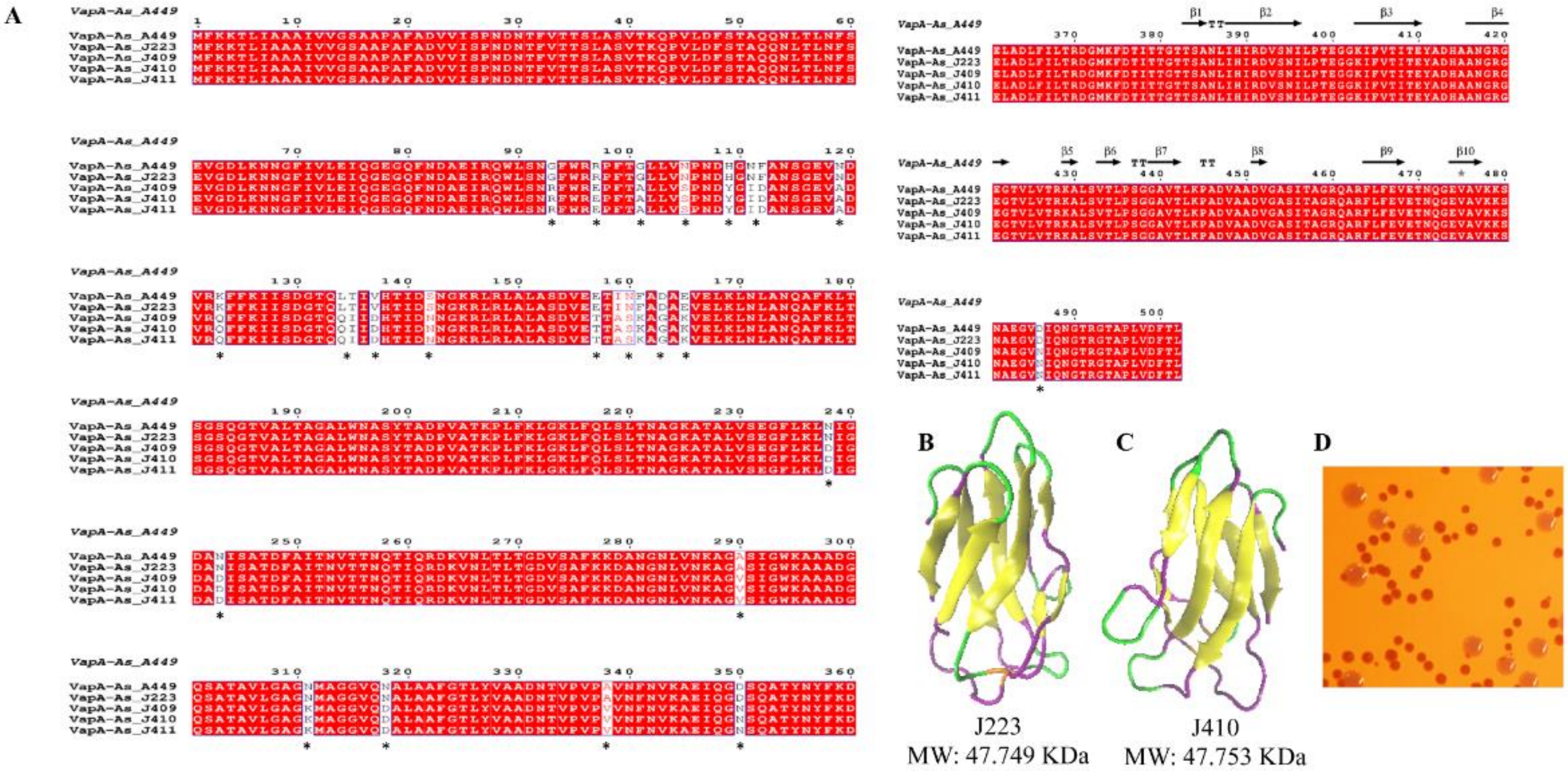

Figure S3. *A. salmonicida* A-layer (VapA) sequence and structure analysis. **A.** Amino acid sequence alignment of VapA protein of typical *A. salmonicda* J223 and A449, and atypical *A. salmonicida* strains J409, J401 and J411. Alignment and secondary structure analysis were performed using Espript3.0 with default parameters. **B.** 3D modeling protein structure of J223-VapA protein. **C.** 3D modeling protein structure of J410-VapA protein. Prediction and 3D modeling analysis was performed using VMD software and A449 –VapA protein structure as reference (.pdb file). Turns (Green), Coils (Purple), Extended beta sheets (Yellow), Bridging beta sheets (Orange). **D.** Red-white colonies of *A. salmonicida* J223 growth at 28° C (17 h) plated onto TSA-congo red plate and incubated at 15° C. Black arrows denote white colonies.
