# Supplementary material for: Comparative Genomics of Typical and Atypical Aeromonas salmonicida Complete Genomes Revealed New Insights into Pathogenesis Evolution
Source: Microorganisms. 2022 Jan 15;10(1):189. doi: 10.3390/microorganisms10010189 (PMC8780938; doi:10.3390/microorganisms10010189)
Supplement: Supplementary file 1 [file microorganisms-10-00189-s001.zip › microorganisms-1512247 supplementary/Supplementary Tables.pdf]

Supplementary Data

Table S1. Enzymatic profile of *A. salmonicida* using the API ZYM system.

Enzyme Assayed for	J409	J410	J411	J223
Alkaline phosphatase	+	+	+	+
Esterase (C_4)	+	-	-	-
Esterase lipase (C_8)	+	+	+	+
Lipase (C_{14})	+	+	+	+
Leucine arylamidase	+	+	-	+
Valine arylamidase	-	+	-	-
Cystine arylamidase	+	-	+	-
Trypsin	+	+	+	-
α-Chymotrypsin	-	+	+	-
Acid Phosphatase	+	+	+	+
Naphthol-AS-BI-Phosphohydrolase	+	+	+	+
α-Galactosidase	-	-	-	-
β-Galactosidase	-	-	-	-
β-Glucoronidase	-	-	-	-
α-Glucosidase	-	-	-	-
β-Glucosidase	-	-	-	+
N-Acetyl-β-glucosaminidase	-	+	-	-
α-Mannosidase	-	-	-	-
α-Fucosidase	-	-	-	-

Table S2. Enzymatic profile of *A. salmonicida* using API20NE.

Enzyme Assayed for	J409	J410	J411	J223
Reduction of nitrates to nitrites	+	+	+	+
Indole production	-	-	-	-
Glucose fermentation	+	+	+	+
Urease	-	-	-	-
β-Galactosidase	+	+	+	-
Hydrolysis of:				
Arginine	+	+	+	+
Esculin	+	+	+	+
Gelatin	+	+	+	+
Assimilation of:				
D-Glucose	+	+	+	+
L-Arabinose	+	+	+	-
D-Mannose	+	+	+	+
D-Mannitol	+	+	+	+
N-acetyl-glucosamine	+	+	+	+
D-maltose	-	-	-	+
Potassium gluconate	+	+	+	-
Capric acid	+	+	+	-
Adipic acid	-	-	-	-
Malic acid	+	+	+	+
Trisodium citrate	+	+	+	-
Phenylacetic acid	-	-	-	-
Code	5577555	5577555	5577555	5554244

Table S3. Enzymatic profile of *A. salmonicida* using the API 20E.

Enzyme Assayed for	J409	J410	J411	J223
β-Galactosidase	+	+	+	-
Indole production	+	+	+	-
Acetoin production	+	+	+	-
Citrate utilization	-	-	-	-
H_2S production	-	-	-	-
Urease	-	-	-	-
Hydrolysis of:				
L-Arginine	+	+	+	+
L-Lysine	+	+	+	-
L-Ornithine	+	+	+	-
L-Tryptophane	-	-	-	
Gelatinase	+	+	+	+
Assimilation of:				
D-Glucose	-	-	-	+
D-Mannitol	-	-	-	+
Inositol	-	-	-	-
D-Sorbitol	-	-	-	-
L-Rhamnose	-	-	-	-
D-Saccharose	+	+	+	-
D-Melibiose	-	-	-	-
D-Amygdaline	-	-	-	+
L-Arabinose	-	-	-	-

Table S4. *A. salmonicida* genomes used in this study.

Strain	Labels	Size (Mb)	Topology	RefSeq ID	INSDC identifier
J409	Chromosome	4.63	Circular	NZ_CP047374.1	CP047374.1
	p1AsJ409	0.1	Circular	NZ_CP047375.1	CP047375.1
J410	Chromosome	4.62	Circular	NZ_CP047376.1	CP047376.1
	p1AsJ410	0.072	Circular	NZ_CP047377.1	CP047377.1
J411	Chromosome	4.63	Circular	NZ_CP052034.1	CP052034.1
	p1AsJ411	0.084	Circular	NZ_CP052035.1	CP052035.1
J223	Chromosome	4.7	Circular	NZ_CP048223.1	CP048223.1
	pASal1	0.006	Circular	NZ_CP048225.1	CP048225.1
	pASal2	0.005	Circular	NZ_CP048226.1	CP048226.1
	pASal3	0.007	Circular	NZ_CP048227.1	CP048227.1
	pASal5	0.177	Circular	NZ_CP048224.1	CP048224.1

Table S5. Summary of the RAST annotation genome results.

	**Atypical *A. salmonicida* genome**					
	**J409**		**J410**		**J411**	
	**Chr**	**p1AsJ490**	**Chr**	**p1AsJ410**	**Chr**	**p1AsJ411**
Genome size	4,632,948 bp	99,881 bp	4,617,865 bp	72,285 bp	4,631,770 bp	88,226 bp
G+C content	58.7 %	53.6%	58.7 %	54.8	58.7 %	54.3 %
N° of Subsystems	363	2	365	3	364	2
N° of CDS	4428	141	4395	104	4849	157
N° of RNAs	146	-	146	-	152	-

	**Typical *A. salmonicida* J223**				
	**Chr**	**pASal1**	**pASal2**	**pASal3**	**pASal5**
Genome size	4,702,618 bp	6,139 bp	5,247 bp	7,021 bp	176,655 bp
G+C content	58.5 %	57.4%	52.2 %	55.3 %	54.8 %
N° of Subsystems	344	-	-	1	5
N° of CDS	4551	11	9	12	238
N° of RNAs	144	-	-	-	-

Table S6. Antibiotic resistance gene identification in atypical *A. salmonicida* strains.

							J409		J410		J411	
**RGI Criteria**	**ARO Term**	**SNP**	**Detection Criteria**	**AMR Gene Family**	**Drug Class**	**Resistance Mechanism**	**% Identity**	**% Length**	**% Identity**	**% Length**	**% Identity**	**% Length**
Strict	adeF	-	protein homolog model	resistance-nodulation-cell division (RND) antibiotic efflux pump	fluoroquinolone antibiotic, tetracycline antibiotic	antibiotic efflux	47.32	97.64	47.32	97.64	46.55	90.56
Strict	Escherichia coli EF-Tu mutants conferring resistance to Pulvomycin	R234F	protein variant model	elfamycin resistant EF-Tu	elfamycin antibiotic	antibiotic target alteration	88.55	96.33	88.55	96.33	88.55	96.33

Gene description	J409	J410	J411
Beta-lactamase	GO992_07995 *	GO994_16895	GO993_12435 *
Subclass B2 metallo-beta-lactamase (*bla*)	GO992_09850<br>GO992_16415 *	GO994_031160 *<br>GO994_18760	GO993_14300 *<br>GO993_20940 *
Class D beta- lactamase (*blaOXA*)	GO992_13085	GO994_22030	GO993_17570

(*) Indicates a pseudo gene.

Table S7. Antibiotic resistance gene identification in typical *A. salmonicida*.

							***A. salmonicida* J223**					
							CP048223.1		CP048225.1	CP048226.1	CP048227.1	CP048224.1
**RGI Criteria**	**ARO Term**	**SNP**	**Detection Criteria**	**AMR Gene Family**	**Drug Class**	**Resistance Mechanism**	**% Identity**	**% Length**	**-**	**-**	**-**	**-**
Strict	*adeF*		protein homolog model	resistance-nodulation-cell division (RND) antibiotic efflux pump	fluoroquinolone antibiotic, tetracycline antibiotic	antibiotic efflux	43.66	98.87	-	-	-	-
Strict	TRU-1		protein homolog model	TRU beta-lactamase	cephalosporin, penam	antibiotic inactivation	73.23	100.00	-	-	-	-
Strict	cphA5		protein homolog model	CphA beta-lactamase	carbapenem	antibiotic inactivation	98.8	99.61	-	-	-	-
Strict	*adeF*		protein homolog model	resistance-nodulation-cell division (RND) antibiotic efflux pump	fluoroquinolone antibiotic, tetracycline antibiotic	antibiotic efflux	47.32	97.64	-	-	-	-
Strict	Escherichia coli EF-Tu mutants conferring resistance to Pulvomycin	R234F	protein variant model	elfamycin resistant EF-Tu	elfamycin antibiotic	antibiotic target alteration	88.55	96.33	-	-	-	-

Gene description	CP048223.1	CP048225.1	CP048226.1	CP048227.1	CP048224.1
ampC	AXA69_001940	-	-	-	-
Class D beta- lactamase (*blaOXA*)	AXA69_007990 *	-	-	-	-
CphA family subclass B2 metallo-beta-lactamase	AXA69_11710	-	-	-	-

(*) Indicates a pseudo gene.

Table S8. Insertion sequence abundance in typical and atypical *A. salmonicida* genomes.

**Insertion Sequences Families**	***A. salmonicida* J409**		***A. salmonicida* J410**		***A. salmonicida* J411**		***A. salmonicida* J223**				
	Chrom.	p1AsJ409	Chrom.	p1AsJ410	Chrom.	p1AsJ411	Chrom.	pAsal1	pAsal2	pAsal3	pAsal5
**IS1**											
ISAs8	1		1		1		1				
Unknown	2		2		2		2				
**IS110**											
Unknown		2		1		1					3
**IS256**											
ISAs3	9	3	9	1	9	1	9				4
**IS3**											
ISAs32	1		1		1		1				
ISAs33							1				
ISAs5							15				2
ISAs6	1		1		1		2				
Unknown	9		9		8		6				3
**IS4**											
Unknown	2		2		2						1
**IS481**											
ISAs19							1				
**IS5**											
ISAs4	174	11	174	8	174	4					
ISAs34							1				
Unknown	3	6	3	2	4	1					2
**IS5/IS182**											
Unknown	5	1	5	1	6	3					
**IS21**											
ISAs29/*istB*		1		1			8				4
Unknown		1		1			8				3
**IS30**											
Unknown		1		1		1					4
**Tn3**											
Unknown		1		1							2
**ISAs1**											
Unknown	47		46		47						
**IS630**											
Unknown	23	1	23	1	23	3	35				3

(*) N° of copies identified for each ISs. (Data accessed Nov. 09th, 2021)
